# *LAPTM5* Downregulation‐Driven VAMP8 Phosphorylation Impairs Autophagosome‐Lysosome Fusion and Aggravates Septic Acute Lung Injury

**DOI:** 10.1002/advs.78024

**Published:** 2026-09-27

**Authors:** Lang Jiang, Qilan Li, Hao Liu, Xuehui Gao, Hongyu Nie, Lang Chen, Mei He, Xuefeng Li, Xiangming Fang, Jiqian Xu, Hao Zhang, Jiahong Xia, You Shang

**Affiliations:** ^1^ Department of Critical Care Medicine Union Hospital Tongji Medical College Huazhong University of Science and Technology Wuhan China; ^2^ Department of Vascular Surgery Traditional Chinese and Western Medicine Hospital of Wuhan Tongji Medical College Huazhong University of Science and Technology Wuhan China; ^3^ State Key Laboratory of Reproductive Medicine and Offspring Health Nanjing Medical University Nanjing China; ^4^ Department of Anesthesiology and Intensive Care The First Affiliated Hospital Zhejiang University School of Medicine Hangzhou China; ^5^ Department of Thoracic Surgery Union Hospital, Tongji Medical College Huazhong University of Science and Technology Wuhan China; ^6^ Department of Cardiovascular Surgery Union Hospital, Tongji Medical College Huazhong University of Science and Technology Wuhan China

**Keywords:** ALI, autophagy, inflammation, LAPTM5, macrophage, sepsis

## Abstract

Sepsis‐induced acute lung injury (ALI) is a lethal inflammatory condition with limited therapeutic options. Macrophage autophagic homeostasis is essential to constrain septic ALI inflammation, though its upstream regulation remains elusive. Here, we report that lysosomal‐associated protein transmembrane 5 (LAPTM5) is markedly downregulated in peripheral blood mononuclear cells (PBMCs) from septic ALI patients and alveolar macrophages (AMs) of septic mice, with its expression correlating inversely with the SOFA score. Macrophage‐specific depletion of *Laptm5* exacerbated mouse ALI symptoms, whereas AAV‐mediated *Laptm5* overexpression yielded diametrically opposite effects. Mechanistically, LPS stimulation promotes FOXB1 nuclear accumulation, which in turn represses the transcription and protein expression of LAPTM5. Furthermore, LAPTM5 acts as a molecular scaffold bridging PGAM5 and VAMP8, mediating PGAM5‐dependent VAMP8 dephosphorylation to promote autophagosome‐lysosome fusion and autophagic flux. This cascade clears damaged mitochondria, thereby mitigating intracellular oxidative stress and inflammation. This study defines macrophage LAPTM5 as a critical autophagy regulator that mitigates septic ALI, providing a potential avenue for future therapeutic exploration.

## Graphical abstract

LAPTM5 functions as a critical molecular scaffold bridging PGAM5 and VAMP to facilitate PGAM5‐mediated dephosphorylation of VAMP8. This cascade promotes autophagosome‐lysosome fusion, restores impaired autophagic flux, and clears damaged mitochondria, thereby mitigating cellular oxidative stress and systemic inflammation. Ultimately, activating this protective pathway significantly alleviates sepsis‐induced acute lung injury in mice.

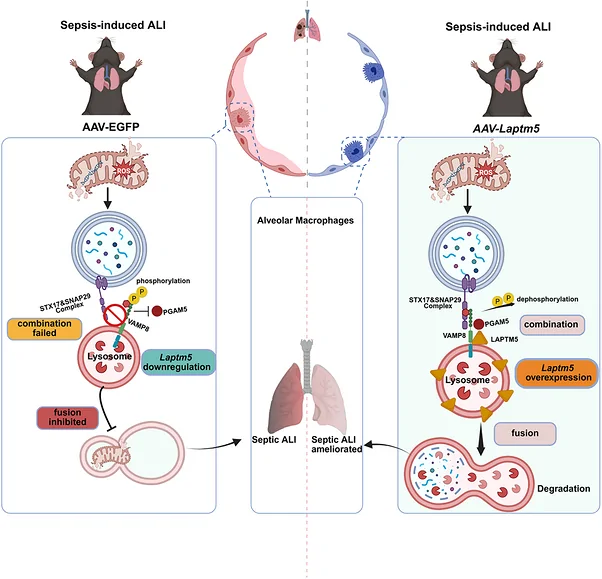

## 1 Introduction

As a life‐threatening multi‐organ dysfunction caused by the host's dysregulated response to infection, sepsis is one of the leading causes of death in ICU, with more than 48 million incident cases and over 11 million sepsis‐related deaths worldwide each year [1, 2]. The lung is the most vulnerable target organ in sepsis, approximately 50% of patients develop acute lung injury/acute respiratory distress syndrome (ALI/ARDS), and the mortality rate rises to 40% once ARDS develops [3, 4]. Unfortunately, the pathogenesis and molecular mechanisms underlying septic ALI remain incompletely elucidated, no targeted therapeutics are currently available in clinical practice, with treatment limited to symptomatic supportive care.

Currently recognized mechanisms of septic ALI include inflammatory imbalance, immunosuppression, mitochondrial damage, and dysregulated autophagy [5]. Sepsis‐related stress causes mitochondrial damage, and imbalanced mitophagy exacerbates intracellular inflammation, which is critically involved in lung injury driven by the cytokine storm and has emerged as a prevailing research frontier [6, 7, 8]. Considering that alveolar macrophages (AMs) account for approximately 95% of intra‐alveolar leukocytes and act as central regulators of pulmonary immunity and inflammation, exploring mitophagy regulatory networks in AMs will be a promising research direction [9, 10, 11].

Lysosomes serve as core organelles in mitophagy regulation, a process in which lysosomal membrane proteins play an indispensable, executionary role. As an important member of the lysosomal transmembrane protein family, LAPTM5 (lysosomal‐associated protein transmembrane 5) localizes primarily to the membranes of lysosomes and late endosomes, playing a unique role in immune regulation, cell surface receptor downregulation, and tumor cell fate determination. Our previous work showed that LAPTM5 recruits CDC42 for lysosomal degradation under high‐fat conditions, suppresses the MAPK pathway, and alleviates NASH, suggesting a potential avenue for future therapeutic exploration [12]. In the meantime, LAPTM5 is essential for preserving lysosomal structural integrity and function [13, 14]. Moreover, previous studies have reported that LAPTM5 facilitates lysosomal degradation of BMPR1A to promote lung‐specific metastasis of renal cancer; it targets WWP2 for lysosomal degradation, leading to PTEN accumulation and B‐cell apoptosis; and it also delivers HIV‐1 envelope glycoproteins to lysosomes for degradation, thus suppressing virion infectivity [15, 16, 17]. Importantly, LAPTM5 serves as a key regulator of inflammatory signaling in macrophages [18]. Collectively, these findings illustrate that LAPTM5 exerts a critical role in regulating lysosome‐associated functions and the autophagic process, which leads us to hypothesize that LAPTM5 may serve as a critical regulator of mitophagy in the context of septic ALI—the hypothesis upon which this study is designed and performed.

In this study, we demonstrated that the protein and mRNA expression of LAPTM5 was significantly downregulated in AMs of septic mice and in the PBMCs of patients with septic ALI. Depletion of LAPTM5 in macrophages markedly exacerbated sepsis‐induced acute lung injury in CLP‐operated mice. Conversely, adeno‐associated virus–mediated macrophage LAPTM5 overexpression effectively ameliorated septic ALI. Furthermore, we found that LAPTM5 reduction was tightly linked to FOXB1‐mediated transcriptional repression, and there exist a negative correlation between LAPTM5 expression and the SOFA score, underscoring its potential association with disease severity. We further observed that the downregulation of LAPTM5 in AMs downregulates mTOR signaling to trigger autophagy induction, while paradoxically impairing autophagic flux. Mechanistically, LAPTM5 acts as an adaptor mediating the interaction between the phosphatase PGAM5 and the lysosomal membrane protein VAMP8. LAPTM5 downregulation impairs PGAM5‐mediated VAMP8 dephosphorylation, which in turn prevents STX17–SNAP29–VAMP8 complex formation, blocks autophagic flux, and ultimately leads to damaged mitochondria accumulation and exaggerated inflammatory responses. Collectively, these findings indicate that LAPTM5 plays a critical regulatory role in septic ALI progression, highlighting its potential as a promising biomarker and therapeutic candidate for this condition.

## 2 Results

### 2.1 LAPTM5 Correlates With the Progression of Septic ALI

**FIGURE 1:**
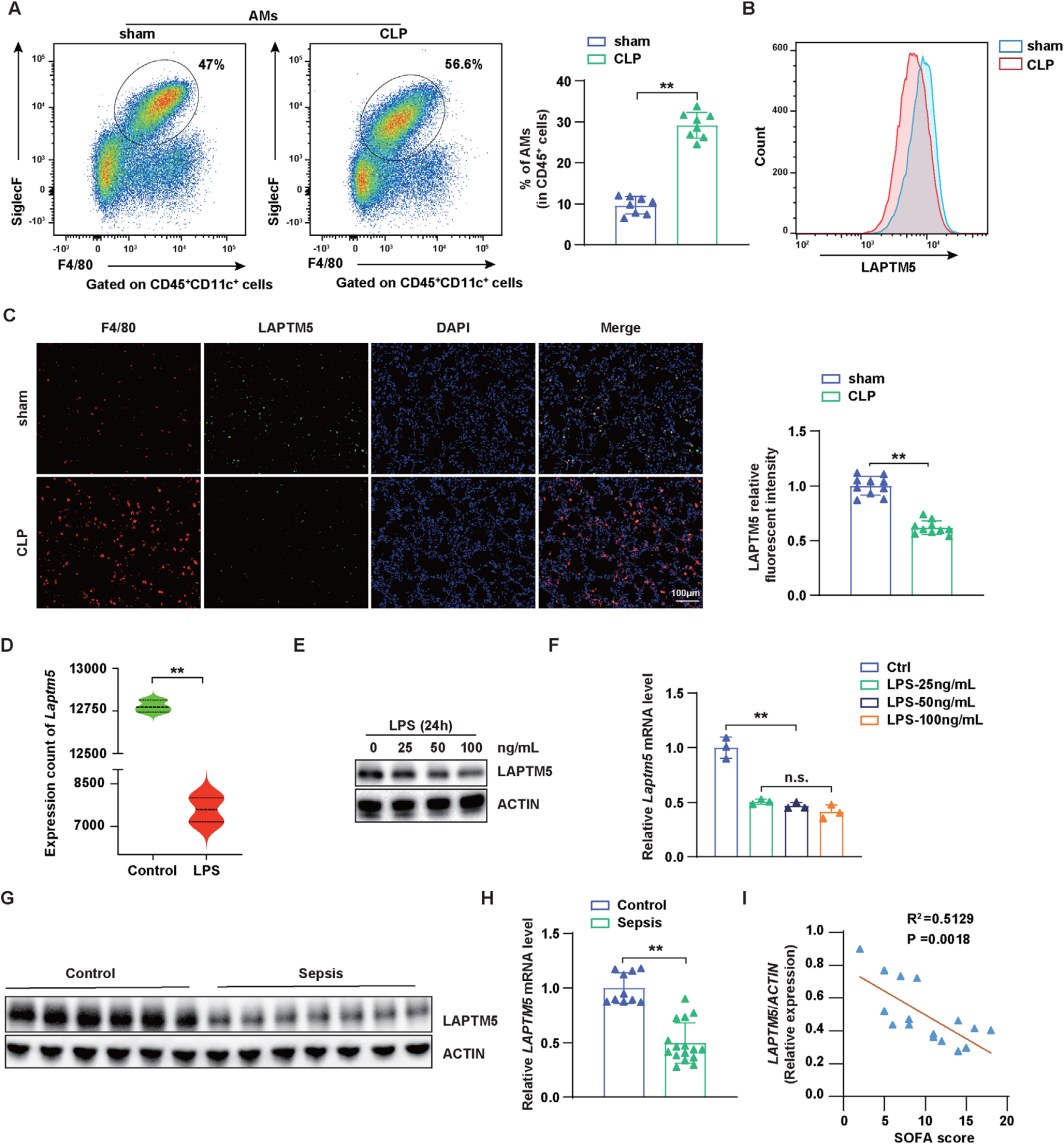
LAPTM5 correlates with the progression of septic ALI. (A). Representative flow cytometry (FCM) plots, cell percentages, and quantitative results of AMs in the lungs of mice subjected to sham or CLP surgery. (B). Flow cytometric detection of LAPTM5 expression in AMs from sham‐ and CLP‐treated mice. (C). Representative immunofluorescence staining and statistical analysis of LAPTM5 (green) in pulmonary macrophages from mouse lung sections of indicated groups. Scale bar, 100 µm, (*n* = 10). (D). Relative *Laptm5* expression in LPS‐treated MH‐S cells based on the GSE277635 dataset. (E‐F). Representative Western blot (E) and RT‐qPCR (F) analyses of LAPTM5 levels in BMDMs from C57BL/6 mice upon LPS stimulation. (*n* = 3 independent experiments). (G). Representative Western blot analysis of LAPTM5 protein levels in human peripheral blood mononuclear cells (PBMCs) from septic (*n* = 7) and non‐septic patients (*n* = 6). (H). RT‐qPCR analysis of *LAPTM5* mRNA levels in human PBMCs from septic (*n* = 16) and non‐septic patients (*n* = 10). (I). Correlation analysis between *LAPTM5* mRNA levels (normalized to β‐actin) and SOFA scores (*p* < 0.01), (*n* = 16). Data are represented as mean ± SD. One‐way ANOVA of Bonferroni post‐hoc test (F) was used to evaluate differences. The two‐tailed Student's *t*‐test was used to evaluate differences in (A, C, D), Mann‐Whitney U test was used to evaluate differences in (H). Simple linear regression was used to evaluate the correlation between *LAPTM5* expression and SOFA scores in (I). Immunoblots are representative of three independent experiments. ^*^ *p* < 0.05, ^**^ *p* < 0.01, n.s., not significant.

Alveolar macrophages (AMs), accounting for 95% of alveolar leukocytes and acting as key regulators of immune and pulmonary inflammation, are critically involved in the pathogenesis of sepsis‐induced acute lung injury (ALI). Given that LAPTM5 is abundantly expressed in lymphoid and myeloid‐derived cells and modulates inflammatory signaling in macrophages, it may play a role in septic ALI [19]. We observed a significant increase in AMs within the bronchoalveolar lavage fluid (BALF) of CLP‐operated mice, accompanied by a marked downregulation of LAPTM5 expression in AMs (Figure 1A,B). Consistent with these findings, immunofluorescence staining of mouse lung tissue sections revealed a substantial reduction in LAPTM5 fluorescence intensity in macrophages following the CLP procedure (Figure 1C). Analysis of public transcriptomic databases further confirmed that *Laptm5* mRNA levels were significantly decreased in MH‐S cells upon LPS challenge (Figure 1D). To validate these in vivo observations, mouse primary macrophages and the MH‐S alveolar macrophage cell line were treated with LPS in a dose‐ and time‐gradient manner. Both protein and mRNA levels of LAPTM5 were consistently downregulated in response to LPS stimulation (Figure 1E,F and Figure S1A–D). Furthermore, clinical relevance was established by examining peripheral blood mononuclear cells (PBMCs) from patients with septic ALI, where LAPTM5 expression at both the protein and mRNA levels was similarly decreased, with its mRNA abundance showing a negative correlation with Sequential Organ Failure Assessment (SOFA) scores (Figure 1G–I). Collectively, the striking inverse correlation between LAPTM5 expression and the progression of septic ALI indicates that LAPTM5 is critically involved in the regulation of this disease.

### 2.2 Macrophage *Laptm5* Downregulation Exacerbates Sepsis‐Induced Inflammation

**FIGURE 2:**
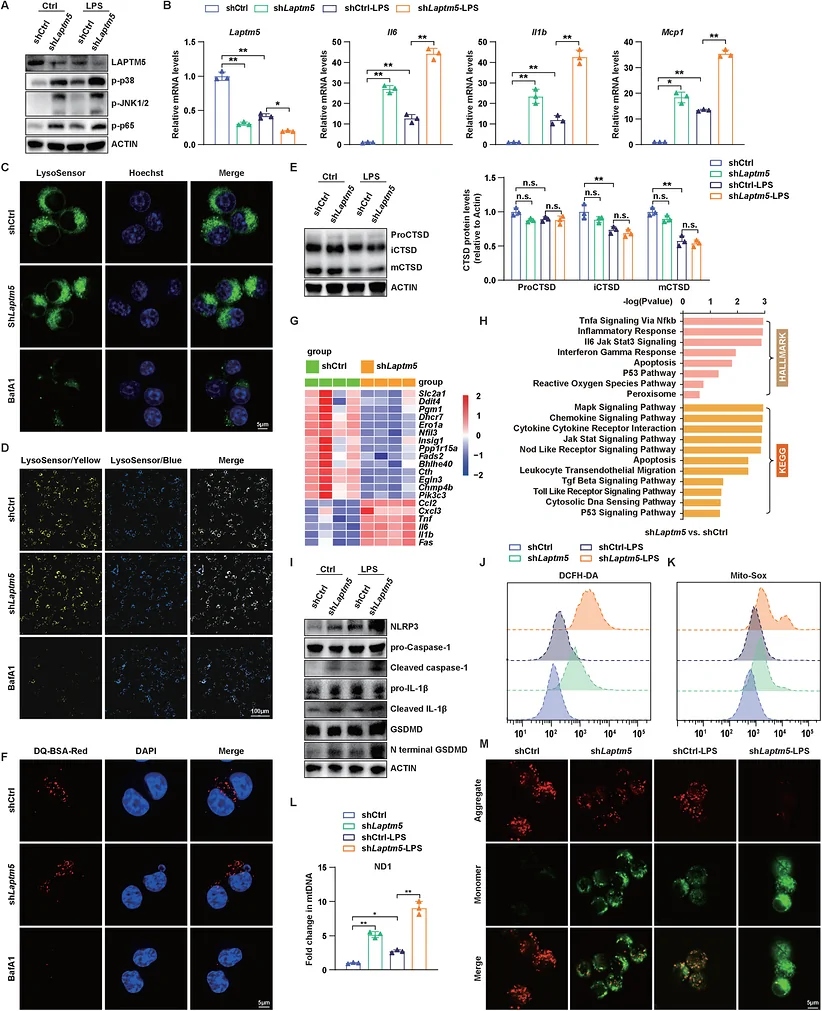
Macrophage *Laptm5* downregulation exacerbates sepsis‐induced inflammation. (A,B). Relative protein (A) and mRNA (B) expression levels of inflammation‐related markers in the indicated group. (*n* = 3 independent experiments). (C). Representative confocal immunofluorescence micrographs and quantitative analysis of LysoSensor Green fluorescence intensity in MH‐S cells. Cells treated with 50 nM BafA1 served as positive controls (Scale bar: 5 µm; quantitative analysis is shown in Figure S2B). (D). Confocal fluorescence micrographs and quantitative analysis of relative acidification levels in MH‐S cells stained with LysoSensor Yellow/Blue DND‐160. Cells treated with 50 nM BafA1 served as positive controls (Scale bar: 100 µm; quantitative analysis is shown in Figure S2C). (E). Immunoblotting analysis of pro‐cathepsin D (ProCTSD), intermediate cathepsin D (iCTSD), and mature CTSD (mCTSD) levels in MH‐S cells from each indicated group, with quantification by densitometric analysis (*n* = 3). (F). Representative confocal fluorescence micrographs and quantitative analysis of DQ‐BSA‐Red fluorescence intensity in MH‐S cells. Cells treated with 50 nM BafA1 served as positive controls (Scale bar: 5 µm; quantitative analysis is shown in Figure S2D). (G,H). Gene expression analysis (G) and Gene Set Enrichment Analysis (GSEA) (H) of differentially expressed genes (DEGs) in *Laptm5*‐knockdown and control MH‐S cells, demonstrating that *Laptm5* deficiency significantly enhances pathways associated with inflammation and apoptosis. (I). Immunoblotting detection of pyroptosis‐related protein markers in each group. (J‐K). Flow cytometric evaluation of intracellular total ROS (DCFH‐DA) (J) and mitochondrial superoxide (Mito‐Sox) (K) levels in each indicated group. (*n* = 3 independent experiments). The quantitative analysis of flow cytometry was shown in Figure S2E. (L). Quantification of mitochondrial DNA (mtDNA) levels in cells from the indicated groups. (*n* = 3 independent experiments). (M). Representative confocal immunofluorescence micrographs of mitochondrial membrane potential (ΔΨm) visualized by JC‐1 staining (red: high potential; green: low potential) and quantification analysis was shown in Figure S2F. Data are represented as mean ± SD. By one‐way ANOVA with Tamhane T2 post‐hoc test for (B‐*Laptm5*, *Mcp1*, D, K, M) and Bonferroni's post‐hoc analysis for (B‐*Il6*, *Il1b*, C, E, F, J, L). Immunoblots are representative of three independent experiments. ^*^ *p* < 0.05, ^**^ *p* < 0.01, n.s., not significant.

Given the significant downregulation of *Laptm5* and its colocalization with macrophages in murine ALI, we postulated that macrophage‐expressed LAPTM5 might be associated with the progression of septic ALI. To test this hypothesis, we generated *Laptm5*‐knockdown MH‐S cells and stimulated them with lipopolysaccharide (LPS) to recapitulate septic ALI in vitro (Figure S2A). Western blotting and RT‐qPCR analyses revealed that *Laptm5* knockdown significantly elevated intracellular inflammatory responses under basal conditions, with further exacerbation following subsequent LPS stimulation (Figure 2A,B). To determine whether LAPTM5 influences lysosomal function, we assessed lysosomal pH using the pH‐sensitive dyes LysoSensor Green and LysoSensor Yellow/Blue (DND‐160), finding that *Laptm5* knockdown did not affect the acidic environment or baseline pH within lysosomes in MH‐S cells (Figure 2C,D and Figure S2B,C). Consistent with this, Western blot analysis revealed no alteration in the levels of mature Cathepsin D (mCTSD)—a key aspartic protease essential for autophagy‐lysosomal homeostasis—confirming that LAPTM5 is dispensable for lysosomal hydrolytic function (Figure 2E) [20]. This was further corroborated by DQ‐BSA‐Red analysis (Figure 2F and Figure S2D). RNA‐sequencing analysis further demonstrated that *Laptm5* knockdown activates biological processes associated with apoptosis, pyroptosis, and inflammation—effects that were further amplified upon LPS challenge (Figure 2G,H and Figure S2G–J). The upregulation of pyroptosis‐related proteins was subsequently verified by Western blotting (Figure 2I). Because mitochondrial dysfunction serves as a central hub linking sepsis‐driven inflammatory cascades to progressive organ failure, we next investigated whether LAPTM5 regulates mitochondrial homeostasis by measuring intracellular and mitochondrial reactive oxygen species (ROS) via flow cytometry. These analyses showed that *Laptm5* knockdown markedly increased both cellular and mitochondrial ROS accumulation (Figure 2J,K and Figure S2E). Furthermore, JC‐1 probe assays evaluating mitochondrial membrane potential and quantification of mitochondrial DNA (mtDNA) release demonstrated that *Laptm5* knockdown enhances intracellular mtDNA leakage and exacerbates mitochondrial membrane potential depolarization, thereby amplifying sepsis‐induced inflammation (Figure 2L,M and Figure S2F).

### 2.3 Macrophage‐Specific Deletion of *Laptm5* Exacerbates Mouse Septic ALI

**FIGURE 3:**
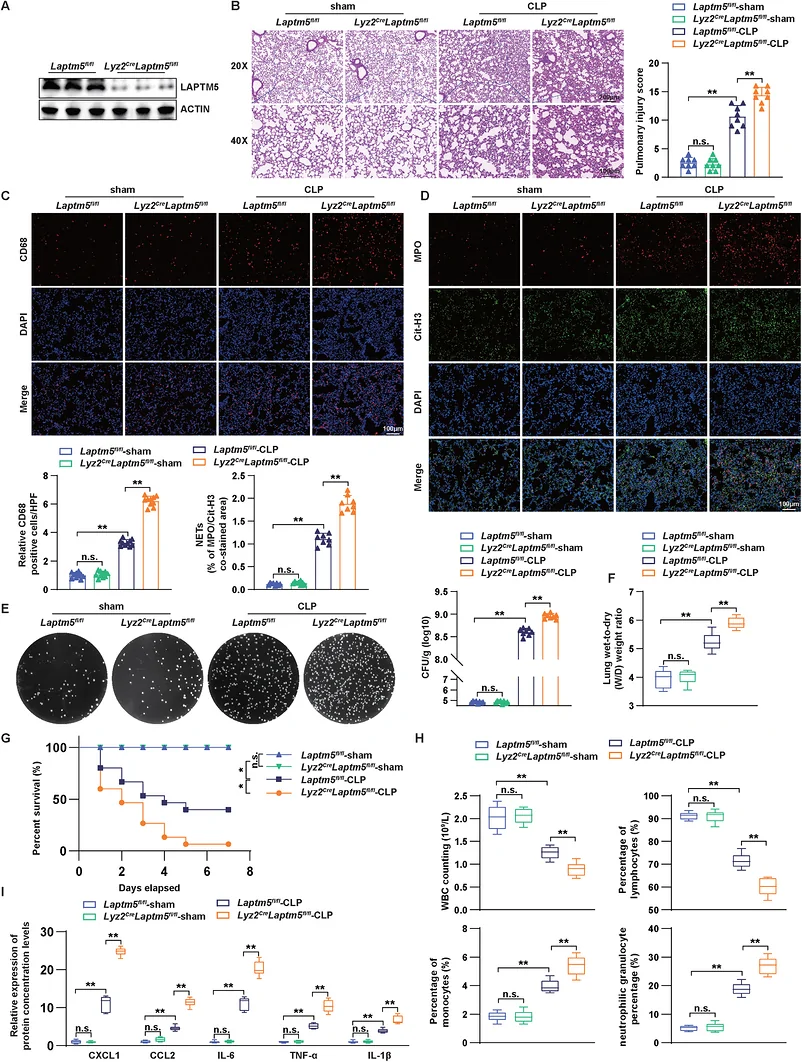
Macrophage‐specific deletion of *Laptm5* exacerbates mouse septic ALI. (A). LAPTM5 protein levels in bone marrow‐derived macrophages (BMDMs) isolated from *Laptm5^fl/fl^* and *Lyz2^Cre^Laptm5^fl/fl^* mice (*n* = 3 mice/group). (B). Representative H&E‐stained lung tissue sections and histological injury scores (*n* = 8). 20X, Scale bar 200 µm. 40 X, Scale bar 100 µm. (C). Representative immunofluorescence images and statistical analysis of CD68 (red) in lung sections from the indicated groups. (*n* = 10). Scale bar, 100 µm. (D). Representative images and quantitative analysis of NETs formation in the lungs, detected via immunofluorescence co‐staining for Cit‐H3 and MPO (*n* = 8). Scale bar, 100 µm. (E). Bacterial loads in murine lungs from the indicated groups. (*n* = 8). (F). Quantifications of the lung wet‐to‐dry ratios from *Laptm5^fl/fl^* and *Lyz2^Cre^Laptm5^fl/fl^* mice 24 h after CLP (*n* = 10). (G). Comparative survival analysis of *Laptm5^fl/fl^* and *Lyz2^Cre^Laptm5^fl/fl^* mice at 7 days after CLP (*n* = 15). (H). Quantitative analysis of peripheral blood routine hematological indicators of *Laptm5^fl/fl^* and *Lyz2^Cre^Laptm5^fl/fl^* mice (*n* = 10). (I). ELISA measurement of inflammatory factors in mouse peripheral blood plasma in the indicated group (*n* = 10). Data are represented as mean ± SD. *P* values were calculated using the Log rank (Mantel–Cox) test (G). By one‐way ANOVA with Tamhane T2 post‐hoc test for (D, H, I) and Bonferroni's post‐hoc analysis for (B, C, E, F). ^*^ *p* < 0.05, ^**^ *p* < 0.01, n.s., not significant.

To further investigate the impact of LAPTM5 on septic ALI and its associated complications, macrophage‐specific *Laptm5* knockout mice were generated by crossing *Laptm5^fl/fl^* and *Lyz2^Cre^* mice (Figure S3A,B). Western blot and RT‐qPCR analyses of BMDMs isolated from *Laptm5^fl/fl^ Lyz2^Cre^* mice confirmed efficient *Laptm5* ablation in macrophages (Figure 3A and Figure S3C). Septic ALI models were established using CLP in both *Laptm5^fl/fl^Lyz2^Cre^* and *Laptm5^fl/fl^* mice to evaluate the functional role of macrophage‐derived LAPTM5 in vivo. Compared with *Laptm5^fl/fl^* controls, *Laptm5^fl/fl^Lyz2^Cre^* mice displayed significantly exacerbated lung injury, characterized by higher histological injury scores and increased inflammatory cell infiltration following CLP, whereas sham‐operated mice of both genotypes exhibited no overt pathological differences (Figure 3B–D). These aggravated pulmonary lesions were accompanied by elevated lung bacterial loads and increased wet‐to‐dry weight ratios (Figure 3E,F). Furthermore, macrophage‐specific deletion of *Laptm5* resulted in a significant reduction in survival rates during sepsis (Figure 3G).

We further analyzed peripheral blood parameters to assess systemic inflammatory responses. Peripheral blood collection and subsequent analyses revealed that during sepsis, *Laptm5^fl/fl^Lyz2^Cre^* mice had a marked reduction in total leukocyte count and lymphocyte proportion, whereas monocyte and neutrophil proportions were significantly increased (Figure 3H). Consistent with these systemic alterations, plasma pro‐inflammatory cytokine levels measured by ELISA were markedly upregulated in *Laptm5^fl/fl^Lyz2^Cre^* mice (Figure 3I). Collectivelly, these findings establish that macrophage‐expressed LAPTM5 plays a critical protective role in mitigating the severity and mortality of sepsis‐induced ALI.

### 2.4 Macrophage *Laptm5* Deficiency Inhibits Autophagic Flux

**FIGURE 4:**
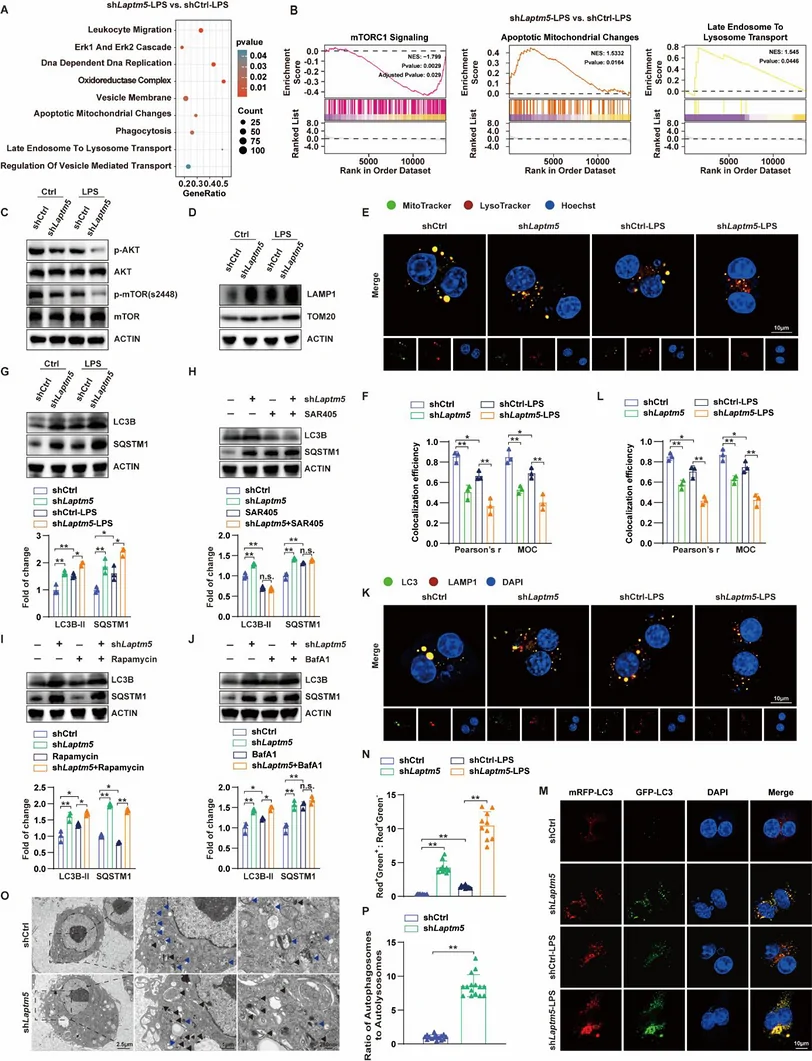
Macrophage *Laptm5* deficiency inhibits autophagic flux. (A,B). Kyoto Encyclopedia of Genes and Genomes (KEGG) enrichment analysis (A) and GSEA of differentially expressed genes (DEGs) (B) in LPS stimulated control and *Laptm5*‐knockdown MH‐S cells. (C,D). Western blot analysis of total and phosphorylated AKT, mTOR, along with LAMP1 and TOM20 expression levels in MH‐S cells from the indicated groups. (E,F). Representative confocal immunofluorescence micrographs and quantitative analysis of MitoTracker Green and LysoTracker Red colocalization in MH‐S cells across the indicated groups (*n* = 3). Scale bar 10 µm. (G). Immunoblotting and densitometric analysis of LC3B and SQSTM1 levels in MH‐S cells from the indicated groups (*n* = 3). (H–J). Immunoblotting assays were performed to assess LC3B and SQSTM1 levels in the absence or presence of SAR405 (H), rapamycin (I) and Bafilomycin A1 (BafA1) (J) in MH‐S cells of indicated groups and quantified by gray scale analysis (*n* = 3). SAR405 (10 µM) for 12 h, rapamycin (10 µM) for 12 h, Bafilomycin A1 (BafA1) (25 nM) for 5 h. (K,L). Representative confocal immunofluorescence micrographs and quantitative analysis of LC3 (green) and LAMP1 (red) colocalization in MH‐S cells across the indicated groups (*n* = 3). Scale bar 10 µm. (M‐N). Representative confocal immunofluorescence micrographs of MH‐S cells transfected with the mRFP‐GFP‐LC3B reporter, alongside quantitative analysis of autophagosome‐to‐ autolysosome ratios in the indicated groups. (*n* = 10). Scale bar 10 µm. (O,P). Transmission electron micrographs of MH‐S cells of indicated groups. The right pictures are the enlarged representations of the boxed regions of the left pictures. Blue arrow indicate autolysosomes; Black arrow indicate autophagosome; Scale bar 2.5 µm (left), 1 µm (middle) and 250 nm (right) (*n* = 15). Data are represented as mean ± SD. By one‐way ANOVA with Tamhane T2 post‐hoc test for (N) and Bonferroni's post‐hoc analysis for (F–J, L). Mann‐Whitney U test was used to evaluate differences in (P).Immunoblots are representative of three independent experiments. ^*^ *p* < 0.05, ^**^ *p* < 0.01. n.s., not significant.

To explore the potential cellular and molecular mechanism by which macrophage LAPTM5 regulates septic ALI, we performed RNA sequencing analysis on control and *Laptm5*‐knockdown MH‐S cells after LPS treatment. KEGG enrichment and Gene Set Enrichment Analysis (GSEA) demonstrated that LAPTM5 expression inversely correlated with pathways associated with autophagy and lysosomal transport, while being closely implicated in oxidative stress and mitochondrial apoptosis (Figure 4A,B and Figure S4A–E). Western blotting confirmed that *Laptm5* knockdown suppressed the mTORC1 signaling pathway, thereby promoting autophagy initiation and the transcription of LAMP1 and TOM20 (Figure 4C,D). However, confocal immunofluorescence microscopy unexpectedly revealed that the colocalization of MitoTracker and LysoTracker decreased following *Laptm5* knockdown and LPS stimulation (Figure 4E,F). Notably, *Laptm5* knockdown concurrently upregulated SQSTM1/p62 and LC3B‐II protein levels, an effect that was further enhanced upon LPS stimulation (Figure 4G). As a selective autophagy receptor constitutively degraded during autophagy, SQSTM1 is widely used to indirectly assess intracellular autophagic flux [21], pointing to a potential blockade caused by *Laptm5* deficiency. To validate this hypothesis, we systematically targeted multiple nodes of the autophagy cascade using SAR405 (an early autophagy inhibitor that suppresses autophagy initiation), bafilomycin A1 (BafA1, a late autophagy inhibitor that impairs lysosomal degradation), or rapamycin (an autophagy inducer) (Figure 4H–J). These pharmacological interventions demonstrated that *Laptm5* downregulation leads to autophagosome accumulation due to impaired autophagic flux rather than affecting the initiation of autophagy, with the severity of this blockade negatively correlating with LAPTM5 expression. Consistent with these findings, *Laptm5* downregulation significantly reduced the confocol fluorescence colocalization between LAMP1 and LC3 (Figure 4K,L).

To corroborate these findings, we performed mRFP‐GFP‐LC3 dual‐fluorescence reporter assays in MH‐S cells. Because GFP fluorescence is quenched in the acidic environment of lysosomes while RFP remains stable, yellow puncta (Red^+^Green^+^) denote autophagosomes, whereas red‐only puncta (Red^+^Green^−^) represent autolysosomes. *Laptm5* knockdown markedly increased intracellular yellow puncta while decreasing red‐only puncta, indicating autophagosome accumulation and a simultaneous increase in the autophagosome‐to‐autolysosome ratio (Figure 4M,N). Transmission electron microscopy further revealed that *Laptm5* knockdown led to a prominent accumulation of double‐membrane autophagosomes and insufficient degradation, compared to the abundant single‐membraned autolysosomes seen under basal conditions (Figure 4O,P). Together, these data demonstrate that *Laptm5* deficiency disrupts the fusion of autophagosomes with lysosomes, resulting in blocked autophagic flux.

### 2.5 Macrophage *Laptm5* Deficiency Promotes the Phosphorylation of VAMP8

**FIGURE 5:**
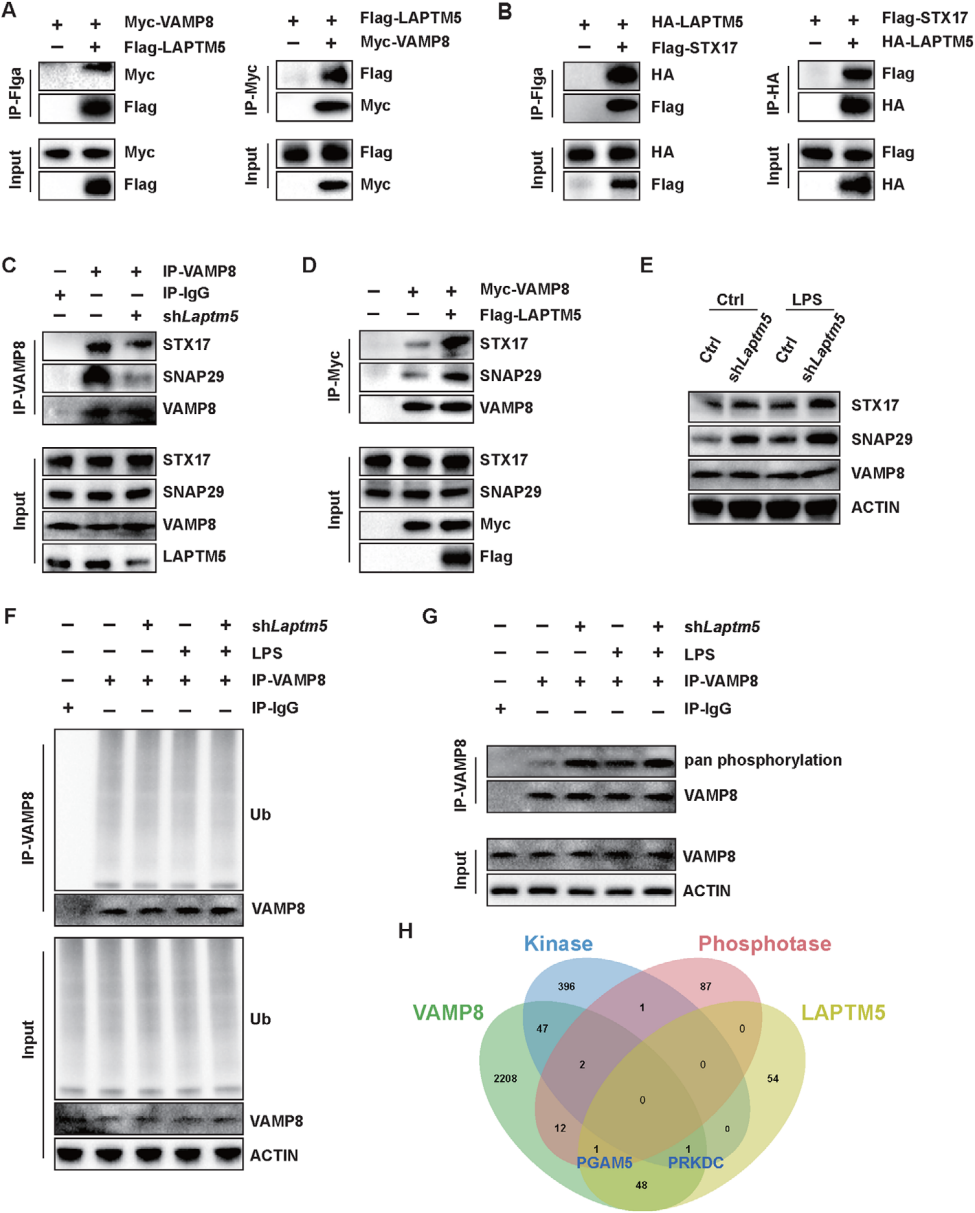
Macrophage *Laptm5* deficiency promotes the phosphorylation of VAMP8. (A). Co‐IP show the interaction between LAPTM5 and VAMP8. (B). Co‐IP shows the interaction between LAPTM5 and STX17. (C,D). Co‐IP analysis evaluating the interactions among STX17, SNAP29, and VAMP8 upon *Laptm5* knockdown (C) and overexpression (D). (E). Western blot analysis of STX17, SNAP29, and VAMP8 expression levels in MH‐S cells from the indicated groups. (F). Co‐IP results show the effect of different treatment on ubiquitination of VAMP8. (G). Co‐IP results show the effect of different treatment on phosphorylation of VAMP8. (H). Venn diagram analysis illustrating the intersection of candidate regulators identified from four databases. Immunoblots are representative of three independent experiments.

The soluble N ethylmaleimide‐sensitive factor attachment protein receptor (SNARE) complex serves as the core machinery mediating the autophagosome‐lysosome fusion process [22]. Specifically, the autophagosome‐associated Qa‐SNARE Syntaxin 17 (STX17) interacts with the Qbc‐SNARE synaptosome‐associated protein 29 (SNAP29) and the lysosome‐localized R‐SNARE vesicle‐associated membrane protein 8 (VAMP8) to form a four‐helix bundle that drives membrane fusion [23]. Co‐immunoprecipitation (Co‐IP) assays revealed that LAPTM5 physically interacts with both VAMP8 and STX17 (Figure 5A,B). *Laptm5* knockdown impaired SNARE complex assembly, whereas its overexpression markedly enhanced the interaction of VAMP8 with STX17 and SNAP29 (Figure 5C,D). Western blot analysis further demonstrated that protein levels of STX17 and SNAP29 were upregulated in *Laptm5*‐deficient cells upon LPS stimulation, whereas VAMP8 abundance remained largely unchanged (Figure 5E). These findings suggest that the elevated expression of STX17 and SNAP29 likely represents a compensatory feedback response to the impaired autolysosomal fusion caused by LAPTM5 deficiency. Given that both LAPTM5 and VAMP8 are resident lysosomal membrane proteins and that VAMP8 does not undergo a similar feedback upregulation, we hypothesized that VAMP8 acts as a key downstream effector through which LAPTM5 modulates autophagosomal‐lysosomal fusion. Previous studies have established that both phosphorylation and ubiquitination regulate VAMP8‐mediated autolysosome formation [24, 25]. Notably, the ubiquitination status of VAMP8 remained unaltered upon changes in LAPTM5 expression, whereas its phosphorylation level was significantly increased following *Laptm5* knockdown (Figure 5F,G). By integrating proteomic profiling of LAPTM5 and VAMP8 with intersect analysis against curated phosphorylation databases, we identified two candidate intermediate regulators linking this pathway: the phosphatase PGAM5 and the protein kinase PRKDC (Figure 5H).

### 2.6 LAPTM5 Mediates PGAM5‐Catalyzed Dephosphorylation of VAMP8

**FIGURE 6:**
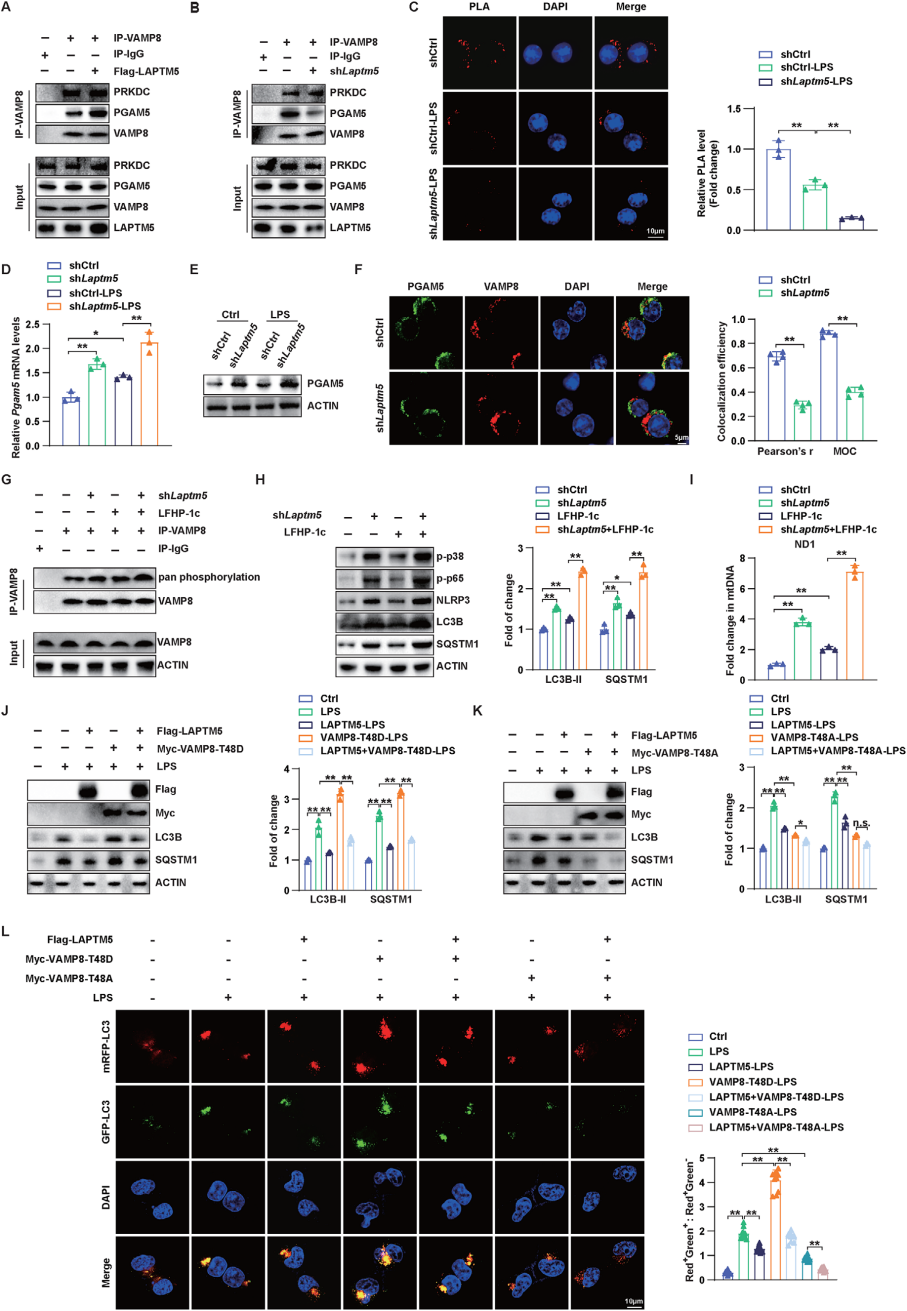
LAPTM5 mediates PGAM5‐catalyzed dephosphorylation of VAMP8. (A,B). Co‐IP analysis demonstrating the interaction among PRKDC/PGAM5 and VAMP8 upon *Laptm5* overexpression (A) and knockdown (B). (C). Proximity ligation assay (PLA) evaluating the interaction between PGAM5 and VAMP8 following *Laptm5* knockdown and LPS stimulation, with corresponding statistical quantification. Scale bar: 10 µm. (N = 3). (D,E). RT‐qCR and Western blot analysis show the mRNA (D) and protein (E) expression levels of PGAM5 in MH‐S cells from the indicated groups. (*n* = 3 independent experiments). (F). Representative confocal immunofluorescence micrographs and colocalization analysis of PGAM5 (green) and VAMP8 (red) in MH‐S cells from the indicated groups (*n* = 3). Scale bar 5 µm. (G). Co‐IP result show the effect of different treatment on phosphorylation of VAMP8. (H). Western blot analysis show the protein expression of p‐p38, p‐p65, NLRP3, LC3B and SQSTM1 in MH‐S cells of the indicated groups and quantified by gray scale analysis (*n* = 3). (I). Detection of mitochondrial DNA (mt‐DNA) levels in MH‐S cells from the indicated groups. (*n* = 3 independent experiments). (J,K). Western blot analysis show the protein expression of LC3B and SQSTM1 in MH‐S cells of the indicated groups and is quantified by gray scale analysis (*n* = 3). (L). Representative confocal immunofluorescence micrographs of 293T cells co‐transfected with the mRFP‐GFP‐LC3B reporter and the indicated plasmids, alongside quantitative analysis of autophagosome‐to‐ autolysosome ratios among the experimental groups. (*n* = 10). Scale bar 10 µm. T48D, phosphomimic; T48A, dephosphorylation mimic. LFHP‐1c, 10 µM, 24 h. Data are represented as mean ± SD. The two‐tailed Student's *t*‐test was used to evaluate differences in (F). By one‐way ANOVA with Tamhane T2 post‐hoc test for (L) and Bonferroni's post‐hoc analysis for (C, D, H‐K). Immunoblots are representative of three independent experiments. ^*^ *p* < 0.05, ^**^ *p* < 0.01, n.s., not significant.

To screen for intermediate factors mediating LAPTM5‐dependent regulation of VAMP8 phosphorylation, Co‐IP assays verified a physical interaction between PGAM5, PRKDC and VAMP8. *Laptm5* knockdown impaired the PGAM5–VAMP8 interaction, whereas its overexpression strengthened this binding; by contrast, the association between VAMP8 and PRKDC remained unaltered by changes in LAPTM5 expression (Figure 6A,B). These findings indicate that *Laptm5* downregulation disrupts the PGAM5–VAMP8 interaction, thereby blunting PGAM5‐catalyzed dephosphorylation and elevating VAMP8 phosphorylation. Proximity ligation assays (PLA) further confirmed this interaction and its reduction following *Laptm5* knockdown and LPS stimulation (Figure 6C). Of note, both mRNA and protein levels of PGAM5 were upregulated upon *Laptm5* knockdown and LPS stimulation, likely representing a compensatory feedback response to the weakened PGAM5 and VAMP8 interaction caused by loss of LAPTM5‐mediated scaffolding (Figure 6D,E). Consistent with these observations, confocol fluorescence colocalization between PGAM5 and VAMP8 was markedly diminished following *Laptm5* knockdown (Figure 6F). To test this mechanism pharmacologically, we utilized the small‐molecule inhibitor LFHP‐1c, which targets PGAM5 to suppress its phosphatase activity [26]. Treatment with LFHP‐1c further elevated VAMP8 phosphorylation (Figure 6G). Moreover, in *Laptm5*‐knockdown cells, LFHP‐1c further blocked autophagic flux, promoted intracellular mtDNA accumulation, and aggravated inflammatory responses (Figure 6H,I), supporting the role of PGAM5 in mediating VAMP8 regulation. To directly assess the functional impact of VAMP8 phosphorylation on autophagy, we constructed VAMP8^T48D^ (phosphomimic) and VAMP8^T48A^ (dephosphorylation mimic) mutant plasmids. Overexpression of phosphomimetic VAMP8^T48D^ mutant aggravated the blockade of autophagic flux, whereas the non‐phosphorylatable mutant VAMP8^T48A^ further restored autophagic flux in cells with ectopic LAPTM5 expression (Figure 6J,K). These phenotypic effects on autophagic flux were further validated using mRFP‐GFP‐LC3 dual‐fluorescence reporter assays (Figure 6L). Together, these results establish that LAPTM5 recruits PGAM5 to facilitate VAMP8 dephosphorylation, which is essential for successful autophagosome‐lysosome fusion and the maintenance of cellular homeostasis.

### 2.7 FOXB1‐Mediated Transcriptional Repression of LAPTM5

**FIGURE 7:**
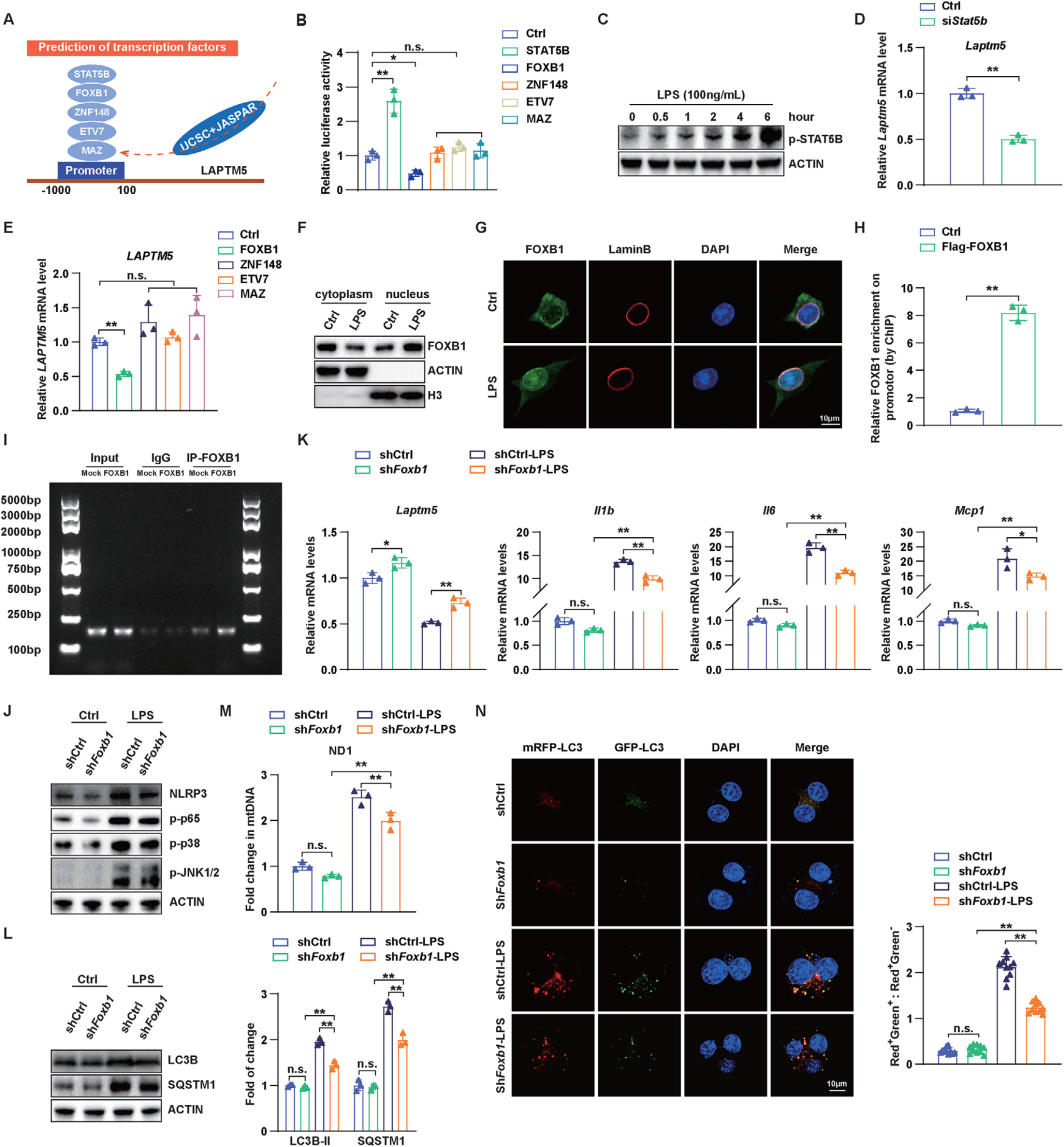
FOXB1‐mediated transcriptional repression of LAPTM5. (A). Schematic diagram illustrating the screening procedure for the identification of key transcription factors. (B). Relative luciferase activity in 293T cells co‐transfected with the LAPTM5‐luciferase reporter plasmid, a Renilla luciferase internal control, and expression plasmids for five candidate transcription factors (STAT5B, FOXB1, ZNF148, ETV7 and MAZ) or an empty vector (Control). (C). Western blot analysis of phosphorylated STAT5B in MH‐S cells under different stimulation durations. (D). Detection of *Laptm5* mRNA expression after *Stat5b* knockdown (n = 3 independent experiments). (E). Detection of *LAPTM5* mRNA expression after *FOXB1*, *ZNF148*, *ETV7*, *MAZ* overexpression (*n* = 3 independent experiments). (F). Nuclear‐cytoplasmic fractionation followed by Western blot detection of FOXB1 expression in nuclear and cytoplasmic fractions. H3 and β‐actin served as nuclear and cytoplasmic internal controls, respectively. (G). Representative confocal immunofluorescence micrographs of the co‐localization of FOXB1 (green) and LaminB (red) in MH‐S cells in the indicated groups. Scale bar, 10 µm. (H,I). ChIP‐qPCR (H) and agarose gel electrophoresis (I) were performed to detect the binding enrichment of FOXB1 at the LAPTM5 gene promoter. IgG and Input were used as negative and internal controls, respectively. (J,K). Relative protein (J) and mRNA (K) expression levels of inflammation‐related markers in each indicated group. (*n* = 3 independent experiments). (L). Western blot analysis show the protein expression of LC3B and SQSTM1 in MH‐S cells of the indicated groups and was quantified by gray scale analysis (*n* = 3). (M). Detection of mitochondrial DNA (mt‐DNA) levels in MH‐S cells from the indicated groups. (*n* = 3 independent experiments). (N). Representative confocal immunofluorescence micrographs of MH‐S cells transfected with the mRFP‐GFP‐LC3B reporter, alongside quantitative analysis of autophagosome‐to‐ autolysosome ratios in the indicated groups. (*n* = 10). Scale bar 10 µm. Data are represented as mean ± SD. The two‐tailed Student's *t*‐test was used to evaluate differences in (D, H). By one‐way ANOVA with Tamhane T2 post‐hoc test for (E, N) and Bonferroni's post‐hoc analysis for (B, K‐M). Immunoblots are representative of three independent experiments. ^*^ *p* < 0.05, ^**^ *p* < 0.01, n.s., not significant.

To further delineate the mechanism underlying LAPTM5 mRNA and protein downregulation in AMs during septic ALI, we integrated analyses from the UCSC and JASPAR databases and identified the top five transcription factors with the highest predicted relevance: STAT5B, FOXB1, ZNF148, ETV7, and MAZ (Figure 7A). Luciferase reporter assays showed that STAT5B significantly increased promoter activity, whereas FOXB1 markedly decreased it; while ZNF148, ETV7, and MAZ showed no notable effect (Figure 7B). Because STAT5B requires phosphorylation and dimerization for nuclear translocation and transcriptional activation [27, 28], we assessed its activation status and found that LPS stimulation progressively elevated STAT5B phosphorylation (Figure 7C). However, siRNA‐mediated *Stat5b* knockdown paradoxically reduced *Laptm5* mRNA levels—contrary to the predicted positive regulatory pattern—thereby excluding STAT5B as the primary upstream transcriptional repressor of LAPTM5 (Figure 7D and Figure S5A). We next constructed overexpression plasmids for the remaining four transcription factors. Notably, only FOXB1 overexpression significantly downregulated *LAPTM5* mRNA levels, identifying FOXB1 as a promising transcriptional regulator (Figure 7E). Subsequent nuclear‐cytoplasmic fractionation and confocal immunofluorescence assays revealed that LPS stimulation induced marked nuclear translocation of FOXB1, whereas the subcellular localizations of ZNF148, ETV7, and MAZ remained unchanged (Figure 7F,G and Figure S5B–D). ChIP‐qPCR assays further confirmed that FOXB1 physically binds to the promoter region of LAPTM5 (Figure 7H,I). To evaluate its functional role, we generated stable *Foxb1*‐knockdown MH‐S cell lines. *Foxb1* downregulation partially rescued *Laptm5* expression and attenuated LPS‐induced inflammation (Figure 7J,K). Concomitantly, *Foxb1* silencing partially restored autophagic flux‐evidenced by decreased SQSTM1 expression and reduced mtDNA levels (Figure 7L,M)‐with these phenotypic improvements in reactive oxygen species (ROS) and autophagic flux further validated by flow cytometry and mRFP‐GFP‐LC3 dual‐fluorescence reporter assays (Figure 7N and Figure S5E). These findings collectively demonstrate that FOXB1 acts as an upstream transcriptional repressor of LAPTM5 during septic ALI, providing a mechanistic link for LAPTM5 downregulation and a potential therapeutic exploration.

### 2.8 AAV‐Mediated *Laptm5* Overexpression in Macrophages Alleviated Mouse Septic ALI

**FIGURE 8:**
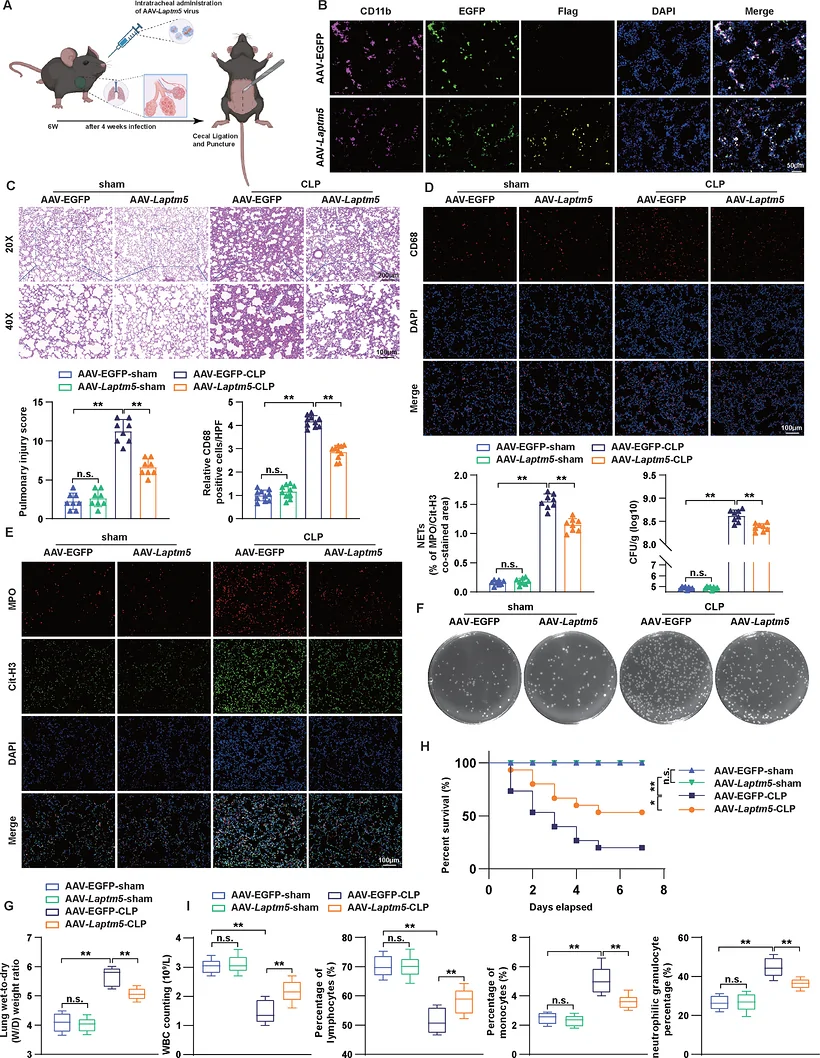
AAV‐mediated *Laptm5* overexpression in macrophages alleviated mouse septic ALI. To generate mice with alveolar macrophage‐specific *Laptm5* overexpression and corresponding controls, C57BL/6J mice received intratracheal instillation of rAAV‐innate(mp)‐Cd11b‐3xFLAG‐*Laptm5*‐2A‐EGFP‐WPRE and rAAV‐innate(mp)‐Cd11b‐EGFP‐WPRE, respectively. Four weeks post‐infection, the mice were subjected to CLP to induce septic ALI. (A). Scheme of constructing AAV‐*Laptm5* mediated septic mice. (B). Immunofluorescence assay was used to validate the successful overexpression of *Laptm5* via AAV delivery in mouse pulmonary macrophages. (C). Representative H&E sections of lung tissues and histological injury scores (*n* = 8). 20X, Scale bar 200 µm. 40 X, Scale bar 100 µm. (D). Immunofluorescence staining and statistical analysis of CD68 (red) in the lung sections of mice in the indicated groups. (*n* = 10). Scale bar, 100 µm. (E). Representative images and quantitative analysis of NETs formation in the lung detected by immunofluorescence staining with Cit‐H3 and MPO (*n* = 8). Scale bar, 100 µm. (F). Bacterial loads in the murine lung in the indicated groups. (*n* = 8). (G). Quantifications of the lung wet‐to‐dry ratio from AAV‐EGFP and AAV‐*Laptm5* mice 24 h after CLP (*n* = 10). (H). Comparative survival analysis of AAV‐EGFP and AAV‐*Laptm5* mice at 7 days after CLP (*n* = 15). (I). Quantitative analysis of peripheral blood routine hematological indicators of AAV‐EGFP and AAV‐*Laptm5* mice (*n* = 10). Data are represented as mean ± SD. *P* values were calculated using the Log rank (Mantel–Cox) test (H). By one‐way ANOVA with Tamhane T2 post‐hoc test for (E, I, lymphocytes) and Bonferroni's post‐hoc analysis for (C, D, F, G, I, WBC, monocytes and neutrophilic granulocyte). ^*^ *p* < 0.05, ^**^ *p* < 0.01, n.s., not significant.

Finally, *Laptm5*‐overexpressing adeno‐associated virus (AAV‐*Laptm5*) was delivered into mouse lungs via intratracheal instillation. Four weeks post‐infection, mice were subjected to CLP to induce septic ALI, with AAV‐EGFP employed as a control vector (Figure 8A). Immunofluorescence analysis of frozen mouse lung sections confirmed the efficient and specific AAV‐mediated overexpression of *Laptm5* in pulmonary macrophages (Figure 8B). Histopathological staining revealed that AAV‐*Laptm5‐*treated mice exhibited significantly attenuated lung injury, as reflected by lower histological injury scores and reduced inflammatory cell infiltration following CLP, whereas sham‐operated groups of both vectors showed no overt pathological differences (Figure 8C–E). These structural improvements were accompanied by decreased lung bacterial loads and reduced lung wet‐to‐dry weight ratios (Figure 8F,G). Furthermore, *Laptm5* overexpression significantly improved survival rates during sepsis (Figure 8H). Analysis of systemic inflammatory parameters demonstrated that AAV‐*Laptm5*‐treated mice experienced a marked increase in total peripheral leukocyte counts and lymphocyte proportions, alongside significantly decreased monocyte and neutrophil proportions during sepsis (Figure 8I). Collectively, these findings highlight LAPTM5 as a promising candidate for therapeutic intervention in septic ALI.

## 3 Discussion

Sepsis‐triggered ALI constitutes a lethal clinical complication with scarce efficacious therapeutic interventions. Excessive and uncontrolled inflammation driven by activated macrophages precipitates systemic multiple organ dysfunction and irreversible tissue damage, serving as the predominant determinant of mortality among septic individuals. In the current investigation, we comprehensively elucidated for the first time the functional implication and molecular regulatory mechanism of LAPTM5 in modulating alveolar macrophages (AMs) autophagy, as well as its role in the pathological progression of septic ALI. Collectively, our empirical evidence identifies LAPTM5 as an indispensable modulator of autophagic flux in AMs and a prospective therapeutic candidate for the management of septic ALI. The pivotal findings are summarized as follows: (1) PBMCs derived from septic patients and AMs isolated from septic mice exhibited pronounced downregulation of LAPTM5. (2) Macrophage‐specific *Laptm5* ablation markedly aggravated sepsis‐provoked pulmonary edema and inflammatory cellular infiltration, accompanied by a prominent elevation in the mortality rate of septic mice. In contrast, targeted overexpression of *Laptm5* in macrophages substantially ameliorated sepsis‐related pulmonary pathological lesions and conferred survival benefits. (3) FOXB1 mediates transcriptional suppression of *Laptm5* under septic pathological conditions. (4) Knockdown of *Laptm5* in AMs abolished PGAM5‐dependent VAMP8 dephosphorylation, consequently restraining autophagic flux, exacerbating hyperinflammatory responses, and accelerating the deterioration of septic ALI.

In the contemporary research framework of septic ALI, multiple cellular subsets, including neutrophils, macrophages, alveolar epithelial cells, pulmonary vascular endothelial cells, and innate lymphoid cells, have been extensively validated to orchestrate the initiation, development and resolution of the disease [29, 30]. Given that AMs represent the dominant leukocyte population residing within the alveolar microenvironment and that LAPTM5 is enriched in myeloid and lymphoid lineage cells, our study prioritized the exploration of LAPTM5 biofunction in AMs. Nonetheless, whether LAPTM5 undergoes dynamic expressional remodeling and executes cell‐type‐specific biological functions throughout septic ALI pathogenesis remains an unresolved scientific issue that necessitates in‐depth mechanistic exploration.

In vitro, LPS administration was adopted to stimulate murine BMDMs and MH‐S cells to recapitulate septic inflammatory conditions, resulting in conspicuous reduction in LAPTM5 abundance. Functional assays demonstrated that *Laptm5* silencing alone augmented intrinsic inflammatory activation in macrophages, and such proinflammatory phenotypes were further potentiated following subsequent LPS challenge. Intriguingly, robust inflammatory activation induced by single‐gene knockdown in the absence of exogenous proinflammatory stimuli was beyond our initial experimental anticipation. Additionally, no discernible histopathological disparities were observed between sham‐operated control groups. We speculate that the absence of phenotypic alterations in sham mice is attributable to endogenous homeostatic compensation and adaptive regulatory mechanisms, thereby highlighting the necessity of further dissecting the latent molecular basis underlying such phenotypic divergence. Furthermore, macrophage *Laptm5* deficiency facilitated the secretion of proinflammatory cytokines, aggravated inflammatory infiltration, alveolar architectural destruction and pulmonary edema, and ultimately increased the mortality of septic mice. Collectively, these observations corroborate that AM‐derived LAPTM5 exerts a crucial protective effect and negatively modulates the progression of septic ALI. Furthermore, while the CLP model employed in this study closely mimics the clinical pathophysiology and multi‐microbial etiology of human sepsis, incorporating complementary approaches—such as the LPS‐induced ALI model—may provide robust cross‐validation, significantly enhancing the generalizability of our in vivo mechanistic claims.

Autophagy has been well‐established to exert a crucial regulatory role in the pathophysiological cascade of sepsis‐induced ALI [31, 32, 33]. Transcriptomic sequencing analysis revealed that LAPTM5 expression is inversely correlated with autophagy and lysosomal trafficking signaling, while being closely associated with oxidative stress imbalance and mitochondrial apoptosis. These transcriptomic signatures indicate that AM‐expressed LAPTM5 likely participates in the inflammatory pathogenesis of septic ALI through autophagy‐associated signaling modulation. Mechanistically, *Laptm5* deficiency led to the intracellular upregulation of LC3B and SQSTM1, accompanied by the accumulation of autophagosomes and insufficient degradation, indicative of impaired autophagic flux in AMs.

As a lysosomal transmembrane protein, LAPTM5 has garnered substantial research attention in recent years. Cumulative studies have verified that LAPTM5 governs the lysosomal degradation of downstream target proteins, thereby remodeling intracellular signaling cascades and regulating diverse physiological and pathological processes. For instance, LAPTM5 facilitates the lysosomal degradation of WWP2 to aggravate renal pathological damage during the progression of CKD [34]. Current research pertaining to autophagy predominantly focuses on superficial phenotypic changes in autophagic activity, whereas the molecular regulatory machinery remains poorly characterized. Although previous studies have suggested an association between LAPTM5 and autophagy regulation, and have documented that ginkgetin modulates macrophage autophagy via inhibiting LAPTM5 ubiquitination [35, 36], our study delves deeper into the specific molecular mechanisms by which LAPTM5 governs the autophagic process. Here, we uncover the critical role of the PGAM5–VAMP8 phosphorylation axis in autophagy regulation for the first time, alongside the pioneering finding that the downregulation of LAPTM5 expression during sepsis is transcriptionally repressed by FOXB1, endowing this study with prominent originality and theoretical significance. These preceding studies provided a robust and reliable foundation that paved the way for our current investigation.

Mechanistic validation demonstrated that LPS stimulation facilitates FOXB1 nuclear translocation, which in turn transcriptionally represses LAPTM5. Nevertheless, the partial upregulation of LAPTM5 expression upon *Foxb1* knockdown suggests that multiple transcription factors are collaboratively involved in its transcriptional regulation. However, comprehensively identifying all transcription factors engaged in regulating LAPTM5 expression during septic ALI is exceptionally complex and practically unfeasible. This represents a limitation of the present study and provides an important avenue for future investigation. In this context, the development of small‐molecule inhibitors targeting FOXB1 may offer an innovative therapeutic strategy for septic ALI.

As for the downstream mechanism, previous studies have highlighted the regulatory role of VAMP8 phosphorylation in membrane fusion dynamics. Chen et al. first demonstrated that VAMP8 phosphorylation disrupts autophagosome‐lysosome fusion and blocks autophagic flux, suggesting its therapeutic potential in temozolomide action [37]. Moreover, Cheng et al. found that indole‐3‐propionic acid (IPA) promotes autophagosome‐lysosome fusion by inhibiting VAMP8 phosphorylation, thereby ameliorating hyperoxia‐induced alveolar arrest [38]. Building upon these findings, we pioneer the extension of the biological mechanism of VAMP8 phosphorylation to sepsis‐induced ALI via a LAPTM5/PGAM5‐dependent pathway. However, the exact upstream molecule responsible for VAMP8 phosphorylation remains elusive to date, presenting a critical open question that warrants further in‐depth investigation.

Although in vivo AAV‐mediated macrophage‐specific *Laptm5* overexpression was successfully achieved, stable exogenous overexpression of LAPTM5 could not be realized in in vitro cultured MH‐S cells. Regrettably, the lack of a druggable LAPTM5‐pathway modulator (e.g., a stabilizing peptide, small molecule, or FOXB1 inhibitor) to validate the therapeutic efficacy in vivo remains a notable limitation of our study. Future research will focus on identifying the core functional peptide domain of LAPTM5 that mediates PGAM5‐VAMP8 binding; the synthesis and functional verification of this key peptide fragment will provide a novel direction for targeted intervention in septic ALI.

Clinically, a significant inverse correlation was identified between LAPTM5 expression levels and SOFA scores in septic patients. This clinical evidence supports the potential of LAPTM5 as a promising biological biomarker for evaluating disease severity, assessing clinical progression and predicting prognostic outcomes in septic populations. However, this analysis relies on a single‐center, cross‐sectional dataset lacking an independent validation cohort or adjustments for potential confounders such as comorbidities and concurrent treatments, which also represents a limitation in our collection and analysis of clinical samples. The accumulation of larger‐scale clinical cohorts, coupled with more meticulous and rigorous classification and scoring systems and the development of a LAPTM5‐based predictive model, will further advance the translational application of our findings. In summary, the current study elucidates the protective role and molecular mechanism of AMs‐intrinsic LAPTM5 in septic ALI, providing a novel theoretical basis and a promising therapeutic avenue for the clinical prevention and treatment of sepsis‐associated acute lung injury.

## 4 Experimental Section

### 4.1 Reagents

Key reagents, antibodies, and primers used in this study are listed in Tables S1–S4 of the Supporting Information.

### 4.2 Mice and Treatment

Male wild‐type (WT) C57BL/6J mice were purchased from Beijing Vital River Laboratory Animal Technology Co., Ltd. (Beijing, China). *Laptm5^flox/flox^* mice and *Lyz2^Cre^* transgenic mice were obtained from Shanghai Model Organisms Center, Inc. (Shanghai, China). All male mice aged 8–12 weeks with a body weight of 22–30 g were raised under specific pathogen‐free conditions. Animals were maintained in a thermostatically controlled environment at 22°C–24°C with 40%–70% humidity and a 12‐hour light–dark cycle, with free access to standard chow and drinking water. Detailed information regarding the sgRNA target sequences for knockout mouse construction and the primers for genotyping macrophage‐specific knockout mice is provided in Tables S1 and S2 of the Supporting Information.

Prior to surgery, mice were anesthetized via intraperitoneal injection of a xylazine–ketamine mixture and fixed in a supine position. The cecal ligation and puncture (CLP) model was established via midline laparotomy. Briefly, the abdominal skin was shaved and disinfected, and a 1‐cm midline incision was made to expose the cecum. The cecum was ligated with 4‐0 silk suture at 1 cm proximal to the distal tip, followed by double puncture using a 20‐gauge needle. A small amount of cecal contents was gently extruded from the puncture sites. The ligated cecum was then repositioned into the abdominal cavity, and the abdominal incision was closed in layers. At 24 h after CLP surgery, mice were euthanized, and tissue samples were harvested for subsequent experimental analyses.

### 4.3 Human Subjects

Patients aged 42 to 79 years with severe pneumonia or sepsis, who were admitted to the Intensive Care Unit of Union Hospital, Huazhong University of Science and Technology, between January 3, 2025, and December 20, 2025, were enrolled in this study. The diagnosis of sepsis was made in accordance with the Sepsis 3.0 criteria [39]. Patients meeting any of the following criteria were excluded from the study: (1) those receiving glucocorticoid therapy; (2) those with autoimmune diseases, granulocytopenia, malignant tumors, acquired immunodeficiency syndrome, or other severe chronic pathologies; (3) those with chronic respiratory diseases (e.g., interstitial lung disease, chronic obstructive pulmonary disease), congestive heart failure, or neurological injuries affecting pulmonary function; and (4) pregnant patients. Written informed consent was obtained from all patients or their legal representatives prior to study participation. Venous blood (5 mL) was collected from each patient within 24 h of admission, followed by centrifugation to separate plasma and monocytes, which were stored at −80°C for subsequent analyses. Healthy volunteers were recruited as the control group from March 1 to March 15, 2024, at Union Hospital, Huazhong University of Science and Technology, and all volunteers signed a written informed consent form.

### 4.4 Plasma and BALF Sample Collection

Mouse blood samples were collected from the cardiac apex using heparinized syringes, and plasma was isolated by centrifugation at 1,000 × g for 10 min. For BALF collection, mice were subjected to tracheotomy followed by tracheal cannulation. Briefly, 1 mL of phosphate‐buffered saline (PBS) was injected into the bronchoalveolar tissue and then aspirated back repeatedly for three cycles. The collected BALF was centrifuged at 420 × g and 4°C for 6 min. The resulting pellet was harvested, resuspended in PBS, and subsequently subjected to fluorescence‐activated cell sorting (FACS) using a BD FACSAria II cell sorter (BD biosciences) to enrichCD45^+^ F4/80^+^ CD11c^+^ mouse AMs.

### 4.5 Cell Culture and Treatment

MH‐S (murine alveolar macrophage cell line) and human embryonic kidney 293T cells were purchased from the Cell Bank of Type Culture Collection, Chinese Academy of Sciences (Shanghai, China). All cell lines were routinely tested for mycoplasma contamination, with consistent negative results. Cells were cultured in high‐glucose DMEM supplemented with 10% fetal bovine serum and 1% penicillin–streptomycin, and maintained at 37°C in a humidified incubator with 5% CO_2_. All cell lines used in this study were passaged fewer than 30 times after resuscitation, and regular mycoplasma detection was performed via PCR. For in vitro inflammation modeling, MH‐S cells were stimulated with 100 ng/mL LPS (Cat# L2880; Sigma‐Aldrich). After 24 h of continuous incubation, cells were collected for subsequent experimental detection and analysis. For cell base assays, image and data files were anonymized or independently evaluated without knowledge of the experimental groups.

### 4.6 Western Blot Analysis

Total protein was extracted from tissues and cells using RIPA lysis buffer. Protein concentration was quantified with the Pierce BCA Protein Assay Kit (Cat# 23225, Thermo Fisher Scientific, Waltham, MA, USA). Equal amounts of protein samples were separated by 10% SDS–PAGE and then electrotransferred onto PVDF membranes. The membranes were blocked with 5% skim milk in TBST, followed by overnight incubation with specific primary antibodies at 4°C. After washing, membranes were incubated with HRP‐conjugated secondary antibodies for 1 h. Target proteins were visualized using an ECL chemiluminescence reagent and captured with a ChemiDoc XRS+ imaging system. All ACTIN loading controls presented in the research figures were obtained from independent assays. Detailed information for antibodies is supplied in Table S4 of the Supporting Information.

### 4.7 Quantitative PCR (qPCR) Analysis

Total mRNA was extracted from animal tissues and cultured cells using TRIzol reagent, and cDNA was synthesized with reverse transcription kits from Vazyme. qPCR was conducted using SYBR Green qPCR master mix in accordance with the manufacturer's protocols. The relative mRNA expression of target genes was normalized to the housekeeping gene *β‐actin*. Detailed information regarding the primer sequences is provided in Table S3 of the Supporting Information.

### 4.8 Immunoprecipitation Assays

Immunoprecipitation (IP) was performed to detect protein–protein interactions. Briefly, HEK293T cells were co‐transfected with the indicated plasmids. At 16–24 h post‐transfection, cells were lysed in ice‐cold 150 mM IP lysis buffer (20 mM Tris–HCl, pH 7.4; 150 mM NaCl; 1 mM EDTA; 1% NP‐40) supplemented with a protease inhibitor cocktail (Cat# 04693132001, Roche, Basel, Switzerland). Cell lysates were centrifuged at 12,000 × g for 10 min, and the resulting supernatants were transferred to new EP tubes. Each supernatant was first pre‐cleared with protein A/G agarose beads (Cat# P0233, LABLEAD Inc., Beijing, China) for 1 h, followed by overnight incubation with the corresponding primary antibodies at 4°C. After centrifugation at 3,500 × g for 5 min, the precipitated beads were washed three times with 300 mM IP lysis buffer and twice with 150 mM IP lysis buffer. Finally, the beads were heated at 95°C in SDS loading buffer for 15 min, and the eluted proteins were separated by SDS–PAGE for subsequent western blot analysis.

### 4.9 Ubiquitination Assays

293T cells co‐transfected with the indicated plasmids were lysed in 80 µL of 150 mM IP lysis buffer supplemented with 10 µL of 10% SDS lysis buffer, followed by heat denaturation at 95°C for 15 min. After cooling, 900 µL of 150 mM IP lysis buffer containing a protease inhibitor cocktail was added to the lysates. The samples were then sonicated and centrifuged at 12,000 × g for 15 min. The resulting supernatants were collected and incubated with the specified antibodies and protein A/G agarose beads for 3 h at 4°C. Following incubation, the beads were harvested by centrifugation at 3,500 × g for 5 min and washed three times with 500 mM IP lysis buffer (20 mM Tris–HCl, pH 7.4; 500 mM NaCl; 1 mM EDTA; 1% NP‐40). Finally, the washed beads were boiled at 95°C in SDS loading buffer for 15 min, and the eluted proteins were separated by SDS–PAGE and analyzed via western blotting, as described above.

### 4.10 Lung Wet‐to‐Dry Ratio

The right lung tissues were rapidly excised and weighed to record the wet weight. Subsequently, the tissues were fully dried in an oven at 65°C for 48 h, and the dry weight was measured. The lung wet/dry weight ratio was calculated to assess pulmonary edema.

### 4.11 HE Staining

Tissue paraffin sections were deparaffinized and stained with hematoxylin for 5 min, followed by brief differentiation in 1% acid ethanol for 2–3 s. After rinsing under running water, sections were counterstained with eosin for 3 min and rinsed again with running water for 3–5 min. Subsequently, the sections were dehydrated with a graded ethanol series and cleared in xylene. Hematoxylin–eosin (HE) staining images were acquired to assess the severity of lung tissue injury according to the observed features [39, 40].

### 4.12 Immunofluorescence Staining

To perform the LAPTM5, CD11b, CD68, F4/80, MPO and Cit‐H3 immunofluorescence staining, paraffin sections of mouse lung tissues underwent antigen retrieval and were then incubated with the indicated primary antibodies overnight at 4°C, followed by incubation with fluorophore‐conjugated secondary antibodies at 37°C for 1 h. For OCT‐embedded lung tissues from AAV‐infected mice, the antibody dilution ratios and incubation times were identical to those used for the paraffin sections. Antibody details are provided in the Supplementary Materials. The immunofluorescence images were scanned uniformly using a slide scanner (3DHISTECH, PANNORAMIC MIDI) and captured by SlideViewer (2.5.0‐rtm).

### 4.13 Confocol Microscopy

Cells were fixed with 4% paraformaldehyde for 15 min, treated with 0.1% Triton X‐100, and blocked with 5% bovine serum albumin for 1 h at 37°C. Subsequently, the cells were incubated with primary antibodies overnight at 4°C, followed by incubation with fluorescently labeled secondary antibodies. Nuclei were then stained with DAPI. Immunofluorescence images were captured using a laser‐scanning confocal microscope (Nikon AX, NIS‐Elements 6.0).

### 4.14 Cell Transfection and Autophagic Flux Analysis

Lentiviral transfection of MH‐S and 293T cells was performed using mRFP‐GFP‐LC3 (GeneChem Co., Ltd., Shanghai, China) following the manufacturer's instructions. After 72 h of transfection, puromycin was added to the culture medium to select for resistant clones. Confocal fluorescence microscopy (Nikon AX, NIS‐Elements 6.0) was used to capture images. Yellow and red fluorescence signals were quantified to monitor the progression of autophagic flux under different experimental conditions.

### 4.15 Bacterial Counting

Mice were humanely euthanized under xylazine–ketamine anesthesia. After thoracotomy, lung tissues were aseptically harvested, weighed, and homogenized in 1 mL of sterile PBS. For the quantification of bacterial colony‐forming units (CFU), 10 µL of lung homogenates were serially diluted 1:10 in sterile PBS. Next, 20 µL of each diluted sample was spread onto LB agar plates. Plates were incubated at 37°C for 24 h, and bacterial colonies were counted to assess pulmonary bacterial burden. Bacterial loads were expressed as CFU per ml.

### 4.16 ELISA

The expression levels of CXCL1, CCL2, IL‐6, IL‐1β and TNF‐α were quantified using a customized ABplex Mouse 5‐Plex ELISA Panel (Cat# RK04321, ABclonal Technology Co., Ltd., Wuhan, China) in accordance with the manufacturer's protocols. The concentrations of inflammatory cytokines were calculated based on the established standard curve.

### 4.17 Plasmid construction and Viral Infection

All overexpression plasmids, including *STX17*, *SNAP29*, *VAMP8* and its mutant (T48D, T48A), as well as the *FOXB1*, *MAZ*, *STAT5B*, *ZNF148*, *ETV7*, together with shRNA plasmids for the knockdown of *Laptm5* and *Foxb1* were synthesized and constructed by Paivi Biosciences Inc (Wuhan, China). We used lipofectamine 3000 reagent (Vazyme, TL301‐01) for plasmids and siRNA transfection. All adeno‐associated viruses (AAVs), including rAAV‐innate(mp)‐Cd11b‐3xFLAG‐*laptm5*‐2a‐EGFP‐WPRE and rAAV‐innate(mp)‐Cd11b‐EGFP‐WPRE were purchased from BrainVTA Co., Ltd. (Wuhan, China) ^[^ 41]. AAVs were stored at ‐80°C until use. Mice were subjected to intratracheal instillation of 20 µL of the virus at a concentration of 5 × 10^^12^ genome copies/mL.

### 4.18 Luciferase Reporter Assay

The human LAPTM5 promoter region was cloned into the pGL3‐Basic vector (Addgene, #212936). For dual‐luciferase reporter assays, 293T cells were co‐transfected with the LAPTM5 promoter reporter plasmid, the pRL‐TK Renilla luciferase internal control, and either an empty vector or expression plasmids encoding STAT5B, FOXB1, MAZ, ZNF148, or ETV7. Following a 48 h incubation, luciferase activity was measured using the Dual‐Luciferase Reporter Assay System (Promega, E2920). Normalized firefly luciferase activity relative to Renilla luciferase is presented as relative luciferase activity.

### 4.19 Proximity Ligation Assay

Proximity ligation assay (PLA) was performed using the Duolink kit (Sigma‐Aldrich, DUO92102) in accordance with the manufacturer's instructions. Briefly, MH‐S cells were fixed with 4% paraformaldehyde for 10–15 min and permeabilized with 0.2% Triton X‐100 for 10 min. Following blocking for 1 h at 37°C, cells were incubated overnight at 4°C with primary antibodies against VAMP8 and PGAM5. After washing, cells were incubated with PLA PLUS and MINUS probes for 1 h at 37°C, followed by ligation for 30 min and rolling‐circle amplification for 100 min at the same temperature. After final washes, coverslips were mounted using an antifade mounting medium containing DAPI. Confocal micrographs were captured and quantified using ImageJ software.

### 4.20 RNA‐seq and Data Processing

RNA‐seq and subsequent data processing were performed using the UID mRNA‐seq system (Seqhealth Technology Co., Ltd., Wuhan, China). Specifically, total RNA was extracted from MH‐S cells, which were divided into control and *Laptm5* knockdown groups and treated with LPS, using TRIzol Reagent (Thermo Fisher Scientific, Cat# 15596026). Following RNA extraction, genomic DNA contamination was eliminated by treating the samples with DNase I (NEB, Cat# M0303L). The purity of the extracted RNA was evaluated by measuring the A260/A280 ratio using a Nanodrop OneC spectrophotometer (Thermo Fisher Scientific). RNA integrity was verified with the LabChip GX Touch system (Revvity). The concentration of qualified RNA samples was determined using a Qubit 3.0 fluorometer and the Qubit RNA Broad Range Assay Kit (Thermo Fisher Scientific, Cat# Q10210). For library construction, total RNA was used as input to prepare RNA sequencing libraries with the KC Digital mRNA Library Prep Kit (Seqhealth Technology Co., Ltd., Wuhan, China) in accordance with the manufacturer's protocols. This kit employs 12‐base random unique molecular identifiers (UIDs) to label individual cDNA molecules, thereby eliminating duplication bias and errors introduced during PCR amplification and sequencing. Library preparation included enrichment of PCR products corresponding to fragments ranging from 200 to 500 base pairs (bp). The enriched libraries were quantified and sequenced on a DNBSEQ‐T7 platform (MGI) using the paired‐end 150 bp (PE150) sequencing mode to generate paired‐end reads. Differentially expressed genes (DEGs) were identified using the R package “edgeR” (version 3.40.2). DEGs with |log2 fold change (FC)| ≥ 1 and p‐value < 0.05 were considered statistically significant.

### 4.21 Gene Set Enrichment Analysis (GSEA)

Gene Set Enrichment Analysis (GSEA) was conducted using the R package “clusterProfiler” (version 4.12.0), with Hallmark, Gene Ontology (GO), and Kyoto Encyclopedia of Genes and Genomes (KEGG) background datasets obtained from the Molecular Signatures Database (MSigDB).

### 4.22 Pathway Enrichment Analysis

Gene Ontology (GO) enrichment analysis of DEGs was also performed using the R package “clusterProfiler” (version 4.12.0), with a p‐value cutoff of 0.05 to define statistically significant enrichment.

### 4.23 Mass Spectrometry Analysis

To screen potential binding partners of VAMP8, 293T cells were transfected with empty Myc‐Vector or Myc‐VAMP8 plasmids for 48 h, and cellular proteins were subsequently harvested for co‐immunoprecipitation (Co‐IP). Precipitated samples were separated by SDS–PAGE and visualized using a Fast Silver Stain Kit (Cat# P0017S, Beyotime). Immunoprecipitation‐mass spectrometry (IP‐MS) analysis was performed by SpecAlly Life Technology Co., Ltd. (Wuhan, China). Mass spectrometry detection was conducted on a timsTOF Pro mass spectrometer (Bruker, USA). Raw MS data were processed with MaxQuant software (Version 2.2.0.0), and protein identification was performed via the built‐in Andromeda search engine. The UniProt human protein sequence database was used for database searching.

### 4.24 Statistical Analysis

All statistical analyses were performed using SPSS 21.0 (SPSS Inc., Chicago, IL, USA), and GraphPad Prism 8 (GraphPad Software, San Diego, CA, USA). The number of mice per group, as well as the number (n) and type of replicates (biological or technical), are specified in the corresponding figure legends. The normality of data distribution was tested using the Shapiro‐Wilk test, and homogeneity of variance was assessed using Levene's test. For comparisons between two groups, the Student's t‐test was used for normally distributed data, while the Mann‐Whitney U test was applied for non‐normally distributed data. For comparisons of three or more groups with a normal distribution, one‐way analysis of variance (ANOVA) was performed, followed by the Bonferroni post‐hoc test for data with homogeneous variance or the Tamhane T2 post‐hoc test for heteroscedastic data. The non‐parametric Kruskal–Wallis test was utilized for comparisons of three or more groups that did not conform to a normal distribution. The *p* value < 0.05 was considered statistically significant. All data are presented as mean ± standard deviation (mean ± SD). Statistical significance was determined based on the following thresholds: ^*^ *p* < 0.05, ^* *^ *p* < 0.01; n.s., not significant. Detailed statistical methods and *p* values are indicated in the corresponding figure legends. All animal experiments were conducted in a blinded manner, and no data were omitted during statistical analysis.

## Author Contributions

Y.S. and J.Xia. designed the experiments. L.J., Q.L. and X.G. carried out the experiments. H.L. performed bioinformatics analysis. H.N., L.C., M.H. and X.L. analyzed the data. Writing – original draft: L.J. Writing – review and editing: X.F., H.Z. and J.Xu.

## Funding

This work was supported by the National Science and Technology Major Project (Grant No. 2023ZD0506504), the National Natural Science Foundation of China (Grant Nos. 82502641, 82402545, 82500579, and 82300550), the China Postdoctoral Science Foundation (Certificate Number: 2026T190565), the Hubei Provincial Key Research and Development Program of China (Grant Nos. 2023BCB091 and 2025BCB004) and the Hubei Provincial Natural Science Foundation of China (Grant No. 2025AFB349).

## Ethics Approvals

The authors bear full accountability for the integrity and accuracy of the entire work, guaranteeing that any inquiries concerning its validity are rigorously investigated and duly resolved. All animal care and experimental procedures were conducted in strict accordance with the *Guide for the Care and Use of Laboratory Animals* issued by the U.S. National Institutes of Health (NIH, eighth Edition, 2011). All animal experimental protocols were reviewed and approved by the Institutional Animal Care and Use Committee of Tongji Medical College, Huazhong University of Science and Technology. The present animal study was ethically approved by the Experimental Animal Ethics Committee of Tongji Medical College (Approval No. 4613). All procedures involving human participants were reviewed and approved by the Ethics Committee of Union Hospital, Huazhong University of Science and Technology (Approval No.: 0766‐01).

## Conflicts of Interest

The authors declare no conflicts of interest.

## Supporting information




**Supporting File**: advs78024‐sup‐0001‐SuppMat.docx.

## Data Availability

All data and code needed to evaluate and reproduce the results in the paper are provided in the paper and/or the Supplementary Information. The RNA‐seq data with MH‐S cells stimulated by LPS are available in Gene Expression Omnibus under GSE277635. The RNA‐seq data generated in this study has been submitted to and deposited in the GEO database with the number GSE331213. For access, please contact the corresponding author for this study. This study did not generate new materials.
