# Supplementary material for: LAPTM5 Downregulation‐Driven VAMP8 Phosphorylation Impairs Autophagosome‐Lysosome Fusion and Aggravates Septic Acute Lung Injury
Source: Adv Sci (Weinh). 2026 Sep 27:e78024. Online ahead of print. doi: 10.1002/advs.78024 (PMC13616297; doi:10.1002/advs.78024)
Supplement: Supplementary file 1 [file ADVS-9999-e78024-s001.docx]

Supporting Information

***LAPTM5* Downregulation-driven VAMP8 Phosphorylation Impairs Autophagosome-lysosome Fusion And Aggravates Septic Acute Lung Injury**

*Lang Jiang^1†^, Qilan Li^1†^, Hao Liu^2†^, Xuehui Gao^1†^, Hongyu Nie^3^ , Lang Chen^1^, Mei He^1^, Xuefeng Li^1^, Xiangming Fang^4^, Jiqian Xu^1*^, Hao Zhang^5*^, Jiahong Xia^6*^, You Shang^1*^*

1. Department of Critical Care Medicine, Union Hospital, Tongji Medical College, Huazhong University of Science and Technology, Wuhan 430022, China;

2. Department of Vascular Surgery, Traditional Chinese and Western Medicine Hospital of Wuhan, Tongji Medical College, Huazhong University of Science and Technology, Wuhan 430022, China;

3. State Key Laboratory of Reproductive Medicine and Offspring Heath, Nanjing Medical University, Nanjing 211166, China;

4. Department of Anesthesiology and Intensive Care, The First Affiliated Hospital, Zhejiang University School of Medicine, Hangzhou 310011, China;

5. Department of Thoracic Surgery, Union Hospital, Tongji Medical College, Huazhong University of Science and Technology, Wuhan 430022, China;

6. Department of Cardiovascular Surgery, Union Hospital, Tongji Medical College, Huazhong University of Science and Technology, Wuhan 430022, China;

†.These authors contributed equally to this work.

*Correspondence to:

Jiqian Xu MD, PHD, Department of Critical Care Medicine, Union Hospital, Tongji Medical College, Huazhong University of Science and Technology, JieFang Road 1277, Wuhan 430022, China. Email: [jiqiaxu@hust.edu.cn](mailto:jiqiaxu@hust.edu.cn);

Hao Zhang MD, PHD, Department of Thoracic Surgery, Union Hospital, Tongji Medical College, Huazhong University of Science and Technology, JieFang Road 1277, Wuhan 430022, China. Email: [zhanghaowhuh@hust.edu.cn](mailto:zhanghaowhuh@hust.edu.cn);

Jiahong Xia MD, PHD, Department of Cardiovascular Surgery, Union Hospital, Tongji Medical College, Huazhong University of Science and Technology, JieFang Road 1277, Wuhan 430022, China. Email: [jiahong.xia@hust.edu.cn](mailto:jiahong.xia@hust.edu.cn); Fax: +86-027-85776364;

You Shang MD, PHD, Department of Critical Care Medicine, Union Hospital, Tongji Medical College, Huazhong University of Science and Technology, JieFang Road 1277, Wuhan 430022, China. Email: [you_shanghust@163.com](mailto:you_shanghust@163.com);

**This PDF file includes:**

Figures. S1 to S5

Tables. S1 to S4

**Figure S1. LPS stimulation downregulates the expression of LAPTM5 in MH-S cells.**

(**A**). Western blot analysis of LAPTM5 protein expression in MH-S cells treated with a concentration gradient of LPS. (**B**). RT-qPCR analysis of *Laptm5* mRNA expression in MH-S cells treated with a concentration gradient of LPS. (n = 3 independent experiments). (**C**). Time-dependent evaluation of LAPTM5 protein levels in LPS-stimulated MH-S cells detected by Western blot. (**D**). Time-dependent evaluation of *Laptm5* mRNA levels in LPS-stimulated MH-S cells detected by RT-qPCR. (n = 3 independent experiments). Data are represented as mean ± SD. By one-way ANOVA with Tamhane T2 post-hoc test for (**B**) and Bonferroni’s post-hoc analysis for (**D**). Immunoblots are representative of three independent experiments. **P* < 0.05, ***P* < 0.01. n.s., not significant.

**Figure S2. *Laptm5* deficiency aggravates inflammation and mitochondrial dysfunction.**

(**A**). Knockdown efficiency of *Laptm5* in MH-S cells determined by RT-qPCR (n = 3 independent experiments). (**B**). Quantitative analysis of fluorescence intensity of MH-S cells stained with LysoSensor Green. (**C**). Quantitative analysis of relative acidification level of MH-S cells stained with Lysosensor Yellow/Blue DND-160. (**D**). Quantitative analysis of relative fluorescence indensity of MH-S cells stained with DQ-BSA-Red. (**E**). Quantitative flow cytometry analysis of DCFH-DA and Mito-Soxin the indicated groups. (**F**). Quantitative analysis of JC-1 aggregate/monomer ratio of MH-S cells across the indicated groups. (**G**). GSEA analysis reveals the downregulation of interferon-α signaling pathway upon *Laptm5* downregulation. (**H-J**). Heatmap, GSEA, and GO enrichment analysis revealed that *Laptm5* deficiency promoted expression changes in genes related to inflammation, ROS, apoptosis, and mitochondrial function. Data are represented as mean ± SD. By one-way ANOVA with Tamhane T2 post-hoc test for (**C**, **E**-Mito-Sox, **F**) and Bonferroni’s post-hoc analysis for (**A**, **B**, **D**, **E**-DCFH-DA). **P* < 0.05, ***P* < 0.01, n.s., not significant.

**Figure S3. Rudimentary diagram of the generation of *Laptm5* macrophage-specific knockout mice.**

(**A-B**)**.** Construction schematic diagram (**A**) and genotyping results (**B**) of the *Laptm5* macrophage-specific knockout mice. (**C**). *Laptm5* mRNA levels in bone marrow-derived macrophages (BMDMs) isolated from *Laptm5^fl/fl^* and *Lyz2^Cre^Laptm5^fl/fl^* mice. (n = 3 independent experiments).

**Figure S4. Knockdown of *Laptm5* promotes autophagy and aggravates oxidative phosphorylation in macrophages.**

(**A-E**). Gene Set Enrichment Analysis (GSEA) was performed to explore the enrichment of functional pathways in macrophages with *Laptm5* knockdown. The Pi3K-Akt-mTOR signaling pathway was downregulated (**A**), while oxidative phosphorylation (**B**), oxidoreductase complex (**C**), vesicle membrane (**D**) and phagocytosis (**E**) were upregulated. Each plot shows the enrichment curve, gene rank distribution, normalized enrichment score (NES) and *P* value.

**Figure S5. Detection of transcriptional regulatory effects of screened molecules.**

(**A**). RT-qPCR analysis of *Stat5b* mRNA expression levels in MH-S cells treated with siRNA. (n = 3 independent experiments). (**B-D**). Nuclear-cytoplasmic fractionation followed by Western blot detection of ZNF148 **(B)**, ETV7 **(C)** and MAZ **(D)** expression in nuclear and cytoplasmic fractions. H3 and β-actin served as nuclear and cytoplasmic internal controls, respectively. (**E**). Flow cytometry was used to detect intracellular total ROS (using DCFH-DA) and mitochondrial superoxide (using MitoSox) levels in each indicated group, alongside quantitative analysis. (n = 3 independent experiments). Data are represented as mean ± SD. By one-way ANOVA with Bonferroni’s post-hoc analysis for (**A**, **E**). **P* < 0.05, ***P* < 0.01. n.s., not significant

**Table S1. sgRNA target sequences for knockout mice construction**

| **sgRNA** | **Target Sequence (5’-3’)** |
| --- | --- |
| sgRNA1 | GTGGCCCGCGGTAAAGTGTC |
| sgRNA2 | GCCAGCCGGGGAAGCAGT |

**Table S2. Primers for genotyping the macrophage specific knockout mice**

| **Primer** | **Primer Sequence (5’-3’)** |
| --- | --- |
| P1 (Mutant) | CCCAGAAATGCCAGATTACG |
| P2 (Common) | CTTGGGCTGCCAGAATTTCTC |
| P3 (WT) | TTACAGTCGGCCAGGCTGAC |

**Table S3. Primers for qPCR**

| **Gene** | **Primer Sequence(5’-3’)** | |
| --- | --- | --- |
| *Laptm5* | F | AGTGGCCTTTATCACCGTGC |
|  | R | TGGCCGAATTCATGTGCTTC |
| *LAPTM5* | F | CCTGAGCCTACTGATCGGC |
|  | R | CAGGCACAGGAGATAGTCCA |
| *Il6* | F | TAGTCCTTCCTACCCCAATTTCC |
|  | R | TTGGTCCTTAGCCACTCCTTC |
| *Il1b* | F | CCGTGGACCTTCCAGGATGA |
|  | R | GGGAACGTCACACACCAGCA |
| *Mcp1* | F | TACAAGAGGATCACCAGCAGC |
|  | R | ACCTTAGGGCAGATGCAGTT |
| mt-*Nd1* | F | CAACATCGAATACGCCGCAG |
|  | R | AATCGGGGGTATGCTGTTCG |
| *Stat5b* | F | GTGAAGCCACAGATCAAGCA |
|  | R | TCGGTATCAAGGACGGAGTC |

**Table S4. Reagents and antibodies**

1.1 **Antibodies**

| **Name** | **Citation** | **Supplier** | **Cat no.** | **Clone no.** |
| --- | --- | --- | --- | --- |
| anti-b-Actin, dil:1/50000 |  | Abclonal | Cat#AC026 |  |
| anti-LAPTM5,dil:1/1000 |  | Abclonal | Cat#A17995 |  |
| anti-LAPTM5,dil:1/200 |  | Biorbyt | Cat#orb184851 |  |
| anti-CD11b, dil: 1/100 |  | Boster | Cat#BM3925 |  |
| Goat anti-mouse IgG (H+L), dil:1/5000 |  | Abclonal | Cat#AS003 |  |
| Goat anti-rabbit IgG (H+L), dil:1/5000 |  | Abclonal | Cat#AS014 |  |
| anti-JNK1/2, dil:1/1000 |  | Cell Signaling Technology | Cat#9258 |  |
| anti-phospho-p38 (Thr180/Tyr182), dil:1/1000 |  | Cell Signaling Technology | Cat#4511 |  |
| anti-HA, dil:1/1000 |  | Cell Signaling Technology | Cat#3724 |  |
| anti-Flag, dil:1/1000 |  | Abclonal | Cat#AE092 |  |
| anti-Myc,dil:1/1000 |  | Cell Signaling Technology | Cat# 2278 |  |
| anti-HA, dil:1/500 |  | MBL | Cat#M180-3 |  |
| anti-Flag, dil:1/500 |  | MBL | Cat#M185 |  |
| anti-Myc, dil:1/500 |  | MBL | Cat#M047-3 |  |
| anti-phospho-p65 (Ser536), dil:1/1000 |  | Cell Signaling Technology | clone 93H1, Cat#3033 |  |
| anti-phospho-mTOR (Ser2448), dil:1/1000 |  | Proteintech | Cat#67778-1-Ig |  |
| anti-VAMP8, dil:1/1000 |  | Proteintech | Cat#15546-1-AP |  |
| anti-STX17, dil:1/1000 |  | Proteintech | Cat#17815-1-AP |  |
| anti-SNAP29, dil:1/1000 |  | Proteintech | Cat#12704-1-AP |  |
| anti-phospho-Stat5(Tyr694), dil:1/1000 |  | Cell Signaling Technology | Cat#4322 |  |
| anti-LC3B, dil:1/1000 |  | Abclonal | Cat#A19665 |  |
| anti-LC3B, dil:1/200 |  | Cell Signaling Technology | Cat#83506 |  |
| anti-LAMP1, dil:1/1000 |  | Abclonal | Cat#A23947 |  |
| anti-LAMP1, dil:1/200 |  | Cell Signaling Technology | Cat#99437 |  |
| anti-Cathepsin D, dil:1/1000 |  | Cell Signaling Technology | Cat#74089 |  |
| anti-DNA-PKcs, dil:1/1000 |  | Proteintech | Cat#19983-1-AP |  |
| anti-PGAM5, dil:1/1000 |  | Proteintech | Cat#28445-1-AP |  |
| anti-PGAM5, dil:1/200 |  | Proteintech | Cat#68116-1-Ig |  |
| anti-Tom20, dil:1/1000 |  | Proteintech | Cat#11802-1-AP |  |

1.2 **Cell lines**

| **Name** | **Citation** | **Supplier** | **Cat no.** | **Passage no.** | **Authentication test method** |
| --- | --- | --- | --- | --- | --- |
| MH-S |  | Type Culture Collection of the Chinese Academy of Sciences | Cat#GNM43 |  |  |
| HEK293T |  | Type Culture Collection of the Chinese Academy of Sciences | Cat#GNHu17 |  |  |

1.3 **The main software and R packages used for data analysis in this study**

| **Name** | **Version** |
| --- | --- |
| Fastq | 0.23.2 |
| STAR | 2.7.6a |
| edegR | 4.2.0 |
| ggplot2 | 3.5.1 |
| clusterProfiler | 4.12.0 |
| pheatmap | 1.0.12 |
| org.Mm.eg.db | 3.19.1 |
| GSEA | 1.2 |
| GSVA | 1.52.2 |
| GSEABase | 1.66.0 |
| GSEAVis | 0.0.5 |
| dplyr | 1.1.4 |
| rlang | 1.1.3 |
| msigdbr | 7.5.1 |

1.4 **Other (e.g. drugs, proteins, vectors etc.)**

| **Name** | **Manufacturer** | **Catalog** |
| --- | --- | --- |
| SAR405 | MCE | Cat#HY-12481 |
| Rapamycin | MCE | Cat#HY-10219 |
| Bafilomycin A1 | MCE | Cat#HY-100558 |
| JC-1 | MCE | Cat#HY-15534 |
| MitoSOX Red | MCE | Cat#HY-D1055 |
| LFHP-1c | MCE | Cat#HY-139598 |
| DCFH-DA | Beyotime | Cat#S0034S |
| Mouse Mitochondrial DNA Copy Number qPCR Kit with SYBR Green | Beyotime | Cat#D8033S |
| Puromycin Dihydrochloride | Beyotime | Cat#ST551 |
| Polybrene | Beyotime | Cat#C0351 |
| Fetal Bovine Serum (FBS) | Bio-One Biotechnology | Cat#F05-001-B160216 |
| Penicillin-Streptomycin | GIBCO | Cat#15140-122 |
| LysoSensor^TM^ Green DND-189 | Yeasen | Cat#HB210811 |
| Sonication ChIP Kit | Abclonal | Cat#RK20258 |
| Hoechst 33258 | Yeasen | Cat#40729ES10 |
| MitoTracker Green FM | MCE | Cat#HY-135056 |
| LysoTracker Red | Yeasen | Cat#719352ES03 |
| Pentobarbital sodium | Sigma-Aldrich | Cat#P3761 |
| DMEM | GIBCO | Cat#C11995500BT |
| Trizol | Sigma-Aldrich | Cat#T9424 |
| Insulin | Thermo Fisher Scientific | Cat#12585-014 |
| Formalin | Thermo Fisher Scientific | Cat#23-305-510 |
| RIPA buffer | Beyotime Biotechnology | Cat#P0013E |
| Triton X-100 | Sigma-Aldrich | Cat#T8787 |
| DAPI | Invitrogen | Cat#S36939 |
| PMSF | Beyotime Biotechnology | Cat#ST506 |
| HiScript III RT SuperMix for qPCR (+gDNA wiper) | Vazyme | Cat#R323-01 |
| ChamQ SYBR qPCR Master Mix | Vazyme | Cat#Q311-02 |
| cOmplete Protease Inhibitor Cocktail | Roche | Cat#04693132001 |
| PhosStop phosphatase inhibitor | Roche | Cat#4906837001 |
| Hematoxylin | Google biology | Cat#G1004 |
| Eosin | BASO | Cat#BA-4024 |
| Tissue-Tek O.C.T. Compound | Servicebio | Cat#4583 |
| BCA protein assay kit | Thermo Fisher Scientific | Cat#23225 |
| PVDF membranes | Millipore | Cat#IPVH00010 |
| LPS | Sigma | Cat#L2630 |
